# Hybrid and SARS-CoV-2-vaccine immunity in kidney transplant recipients

**DOI:** 10.1016/j.ebiom.2023.104833

**Published:** 2023-10-14

**Authors:** Hassen Kared, Amin Alirezaylavasani, Katrine Persgård Lund, Adity Chopra, Lisa Tietze, Taissa de Matos Kasahara, Guro Løvik Goll, Gunnveig Grødeland, Mari Kaarbø, Anna Varberg Reisæter, Markus Hovd, Kristian Heldal, John Torgils Vaage, Fridtjof Lund-Johansen, Karsten Midtvedt, Anders Åsberg, Ludvig A. Munthe

**Affiliations:** a KG Jebsen Centre for B Cell Malignancies, University of Oslo, Oslo, Norway; b Institute of Clinical Medicine, University of Oslo, Oslo, Norway; c Department of Immunology, Oslo University Hospital, Oslo, Norway; d ImmunoLingo Convergence Center, Institute of Clinical Medicine, University of Oslo, Oslo, Norway; e Division of Rheumatology and Research, Diakonhjemmet Hospital, Oslo, Norway; f Department of Microbiology, Oslo University Hospital, Oslo, Norway; g Department of Transplantation Medicine, Oslo University Hospital-Rikshospitalet, Oslo, Norway; h Norwegian Renal Registry, Oslo University Hospital-Rikshospitalet, Oslo, Norway; i Department of Pharmacy, University of Oslo, Oslo, Norway

**Keywords:** Kidney transplant recipients, COVID-19 vaccination, Anti-viral T and B cell immunity, T cell responses, B cell differentiation and immunity

## Abstract

**Background:**

Kidney transplant recipients (KTR) are at high risk for severe COVID-19 and have demonstrated poor response to vaccination, making it unclear whether successive vaccinations offer immunity and protection.

**Methods:**

We conducted a serologically guided interventional study where KTR patients that failed to seroconvert were revaccinated and also monitored seroconversion of KTR following the Norwegian vaccination program. We analysed IgG anti-RBD Spike responses from dose 2 (n = 432) up to after the 6th (n = 37) mRNA vaccine dose. The frequency and phenotype of Spike-specific T and B cell responses were assessed in the interventional cohort after 3–4 vaccine doses (n = 30). Additionally, we evaluated the Specific T and B cell response to breakthrough infection (n = 32), measured inflammatory cytokines and broadly cross-neutralizing antibodies, and defined the incidence of COVID-19-related hospitalizations and deaths. The Norwegian KTR cohort has a male dominance (2323 males, 1297 females), PBMC were collected from 114 male and 78 female donors.

**Findings:**

After vaccine dose 3, most KTR developed Spike-specific T cell responses but had significantly reduced Spike-binding B cells and few memory cells. The B cell response included a cross-reactive subset that could bind Omicron VOC, which expanded after breakthrough infection (BTI) and gave rise to a memory IgG^+^ B cell response. After BTI, KTR had increased Spike-specific T cells, emergent non-Spike T and B cell responses, and a systemic inflammatory signature. Late seroconversion occurred after doses 5–6, but 38% (14/37) of KTR had no detectable immunity even after multiple vaccine doses.

**Interpretation:**

Boosting vaccination can induce Spike-specific immunity that may expand in breakthrough infections highlighting the benefit of vaccination to protect this vulnerable population.

**Funding:**

10.13039/100016302CEPI and internal funds.

**Research in context**

**Evidence before this study**

We conducted a search of Pubmed and Google Scholar for publications prior to April 2023 relating to organ transplanted patients and kidney transplant recipients combining searches with terms for COVID-19, COVID-19 vaccination, and breakthrough infection. Before vaccination SARS-CoV-2 infection was associated with a high risk of severe COVID-19 in KTR. KTR had dramatically reduced serological responses to three doses of monovalent SARS-CoV-2 vaccines and KTR did not develop RBD-binding IgG^+^ B cells after standard vaccination. KTR also had reduced vaccine-induced T-cell responses after standard vaccination.

**Added value of this study**

This study reveals the gradual increases in vaccine responses with each dose and analysing all facets of immunity, including serological responses, phenotyping of specific and cross-reactive B cell responses, and specific T cell responses after vaccination and breakthrough infection. The study reveals that vaccine-driven B cell responses matured during breakthrough infection, providing cross-reactive neutralizing antibodies, while vaccine-driven T cell responses were more normal and expanded during breakthrough infection. KTR who experienced breakthrough infections had increased inflammatory cytokines that were associated with the conversion from immature B cell responses to fully differentiated IgG + B cell subsets. Despite vaccination, some KTR patients still developed severe COVID-19. However, further vaccination (5th and 6th dose) allowed for responses in a third of patients in each successive vaccination, even for non-responders after the first four doses.

**Implications of all the available evidence**

Vaccination can support specific immune responses that provide Spike-specific immunity in breakthrough infections and highlights the need for continued vaccination in KTR.

## Introduction

SARS-CoV-2 infection is associated with a high risk of severe COVID-19 in kidney transplant recipients (KTR). A meta-analysis based on 74 studies published before January 18th, 2021, and with 5559 KTR showed that SARS-CoV-2 infection caused 23% mortality (95% CI: 21%–27%) and acute kidney injury in 50% of infected patients (95% CI: 44%–56%).^1^

Immunosuppressive drugs such as the calcineurin inhibitors CNI (cyclosporine and tacrolimus), the mammalian target of rapamycin (mTOR)—inhibitors (rapamycin, everolimus) and Inosine Monophosphate Dehydrogenase Inhibitors (mycophenolate acids, MMF) and corticosteroids are used in KTR. These drugs inhibit immune responses in T cells^2^ including T_FH_ cells,^3^ B cells,^4^^,^^5^ as well as the germinal centre reaction and generation of high-affinity isotype, switched antibodies.^6^ They also inhibit the development of alloreactive T cell responses as well as high-affinity anti-donor HLA or anti-alloantigen antibodies. These same immune responses are necessary for T cell-mediated defence against viral infection and the development of virus-neutralizing antibodies.

We and others have demonstrated that after three doses of monovalent vaccine, KTR has reduced serological responses to SARS-CoV-2 vaccines, including reduced levels of neutralizing IgG anti-RBD.^7^, ^8^, ^9^, ^10^, ^11^, ^12^, ^13^, ^14^, ^15^, ^16^, ^17^, ^18^, ^19^, ^20^ Correspondingly, KTR did not develop RBD-binding IgG ^+^ B cells after standard vaccination.^21^ The poor responsiveness has also included reports of reduced vaccine-induced T-cell responses.^9^^,^^10^^,^^22^

We here followed two different KTR cohorts. Firstly, participants from the national registry of 3600 KTR were enrolled in a serologically guided interventional trial to receive 3rd, 4th, or more doses of an mRNA vaccine against SARS-CoV-2 if serological responses were less than 200 BAU/mL, Supplementary Fig. S1a, Supplementary Table S1. Secondly, samples were collected from the national KTR registry cohort that followed the Norwegian SARS-CoV-2 vaccination program. These patients were also vaccinated with doses 3 and 4 (a few months later than the intervention cohort)—regardless of previous serological responses. The main objective of this study was to evaluate the serological responsiveness of KTR to boosting doses of the COVID-19 vaccine. Next, we aimed to quantify and characterize SARS-CoV-2 specific T and B cell immunity in the interventional cohort elicited by repeated mRNA vaccination, with an emphasis on the benefit of additional doses in poor serological responders. Further, we aimed to characterize vaccine-driven T and B cell responses after breakthrough infection.

## Methods

### Patients and vaccinations

**Fig. 1:**
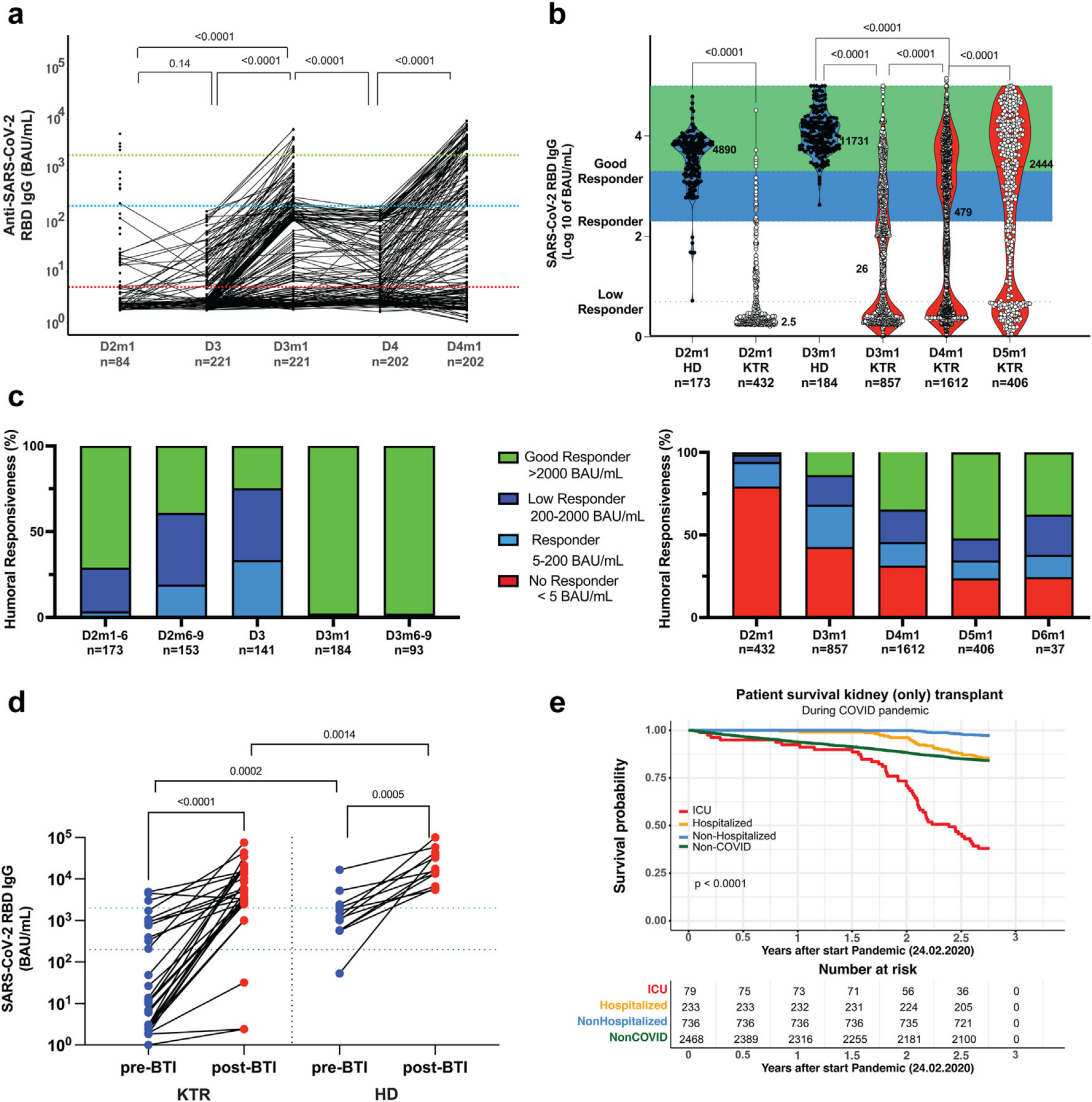
**Serological response in KTR patients and controls after vaccination and BTI**. a. Kidney transplant patients (KTR) vaccinated with mRNA vaccines either BNT162b2 (Pfizer-BioNTech or mRNA-1273 (Moderna), interventional trial (EUDRACT 2021-003618-37, see Methods). Patients with a failed serological response (<200 BAU/mL) were revaccinated with a 3rd dose or also 4th dose. IgG anti-RBD (BAU/mL) 1 month (mo) after dose 2 (D2m1), in blood sample before dose 3 vaccination (D3), 1 mo after dose 3 (D3m1), before dose 4 (D4) and 1 mo after dose 4 (D4m1). b. Comparison of IgG anti-RBD in the healthy donor population (HD) vs KTR 1 mo after doses 2, 3 and in HD 1 mo after dose 3 vs KTR 1 mo after dose 4; and dose 5. Dotted lines for 5, 200, and 2000 BAU/mL separate low responders, responders, and good responders. Violin plots and medians are shown. Two-tailed Mann–Whitney test signed rank test was used. c. IgG anti-RBD in KTR (left) vs normal population (right) in percentage stacked bar chart for vaccination stage as indicated. The number of samples is indicated. d. IgG anti-RBD in KTR (left) vs normal population (right) before and after breakthrough infection. Two-tailed Wilcoxon matched-pairs signed rank test (within the groups) and Mann–Whitney test (between groups); significant p-values are indicated. e. Kaplan Meier patient survival analysis of the entire Norwegian KTR patient cohort alive with a functioning graft by February 24th, 2020 (n = 3748), that during the pandemic (through October 2022) was reported with SARS-CoV-2 infection to the Norwegian Renal Registry. The analysis is grouped by COVID severity, i.e. infection without- or with hospitalization, and also transferred to the ICU department. The log-rank statistical test result is shown in the figure.

In an interventional study (EUDRACT 2021-003618-37),^19^^,^^20^ we invited KTR with no or low serological responses to receive further vaccination. Initially, we chose a cut-off of 10% IgG anti-RBD of levels in the normal population, but this was changed to responses <200 BAU/mL, 1 month after previous mRNA vaccine doses (after standardization allowed WHO units). KTR received the third or fourth vaccination with BNT162b2 (Pfizer-BioNTech) or mRNA-1273 (Moderna). PBMC samples were collected from KTR after doses 2, 3, and 4 (n = 223), and after BTI (n = 32), only plasma was collected from patients receiving doses 5 (n = 406) and 6 (n = 37). In a related observational study, we followed a longitudinal KTR cohort that followed the Norwegian Corona Vaccination program. Here, KTR received 3rd dose and 4th dose somewhat delayed from the interventional study. See Supplementary Table S1 and Fig. 1 for cohort details. Information on the sex of participants was collected as part of the national Id-number, both sexes were included without any preference.

### Study approval

The studies were approved according to the research regulations in Norway, performed according to the Helsinki Declaration, and all patients provided written informed consent. (Approval numbers: REK 2021.233704; REK 2020.135924; REK 2021.229359).

### Sample preparation and HLA typing

KTR were pre-typed in connection with transplantation. Frozen PBMCs from individuals positive for specific HLA were subsequently stained with the corresponding Dextramers/Tetramers Class I restricted (HLA-A∗01:01, HLA-A∗02:01, HLA-A∗24:02, and HLA-B∗07:02) as described.^23^ The non-SARS-CoV-2 related viruses were used as an internal control for the same individuals (CMV, EBV, Influenza, see Supplementary Methods and Supplementary Table S2).

### Flow cytometry

Cryo-preserved PBMCs were enriched for live cells by magnetic depletion of dead cells (Dead cells removal microbeads, Miltenyi) in presence of Citrate buffer and stained with antibody panels to quantify and characterize the phenotype of specific T cell responses directed against Spike peptides (two million cells per sample) and B cell responses to RBD or Spike protein (one million cells per sample). Cells were acquired on a BD FACSymphony (BD Biosciences) or Attune NxT (ThermoFisher). MAbs and stains were as described,^23^ see Supplementary Methods. Statistical significance with thresholds was set: ∗p < 0.05, ∗∗p < 0.01, and ∗∗∗p < 0.001.

### In vitro stimulation assays

Thawed cells were stimulated for 16 h with SARS-CoV-2 PepTivator Spike protein peptides consisting of 15-mer sequences with 11 amino acid overlaps (Wuhan-Hu-1, i.e. wild type WT. Miltenyi Biotec) as described,^23^ see Methods for details. For non-Spike (WT) responses cells were stimulated with Nucleoprotein (PepTivator SARS-CoV-2 Prot N) and Membrane protein (PepTivator SARS-CoV-2 Prot M), consisting of 15-mer sequences with 11 amino acid overlaps in addition to the 4 ORF1ab/Orf3a peptides in Supplementary Table S2 i.e. stimulated with M + N + O. Alternatively, cells were stimulated with 88 pooled WT immunodominant oligopeptides from the whole proteome (PepTivator SARS-CoV-2 Select, Miltenyi Biotec) consisting of peptides from structural proteins (S, M, N) as well as non-structural proteins (O).

### Detection of specific memory CD8 T cells

Antigen-specific CD8 T cells were detected by peptide: HLA multimers as described^23^ (see Supplementary Methods and Supplementary Table S2 for an overview). The peptides are referenced individually in the Supplementary Section.

### Detection of SARS-CoV-2 specific memory B cells

Spike-specific B cells were detected using either sequential staining of biotinylated Recombinant WT SARS-CoV-2 Spike-Trimer and RBD (HEK) (Miltenyi) conjugated with streptavidin-PE or streptavidin-BV786 respectively were combined with probes already conjugated with Alexa Fluor 647 for Spike RBD B1.1, 529 (AFR11056, Gly339Asp, Ser371Leu, Ser373Pro, Ser375Phe, Lys417Asn, Asn440Lys, Gly446Ser, Ser477Asn, Thr478Lys, Glu484Ala, Gln493Arg, Gly496Ser, Gln498Arg, Asn501Tyr, Tyr505His, R&D Systems) and conjugated with Alexa Fluor 488 for Full-length spike B1.1, 529 protein (AFG11061, Ala67Val, His69del, Val70del, Thr95Ile, Gly142Asp, Val143del, Tyr144del, Tyr145del, Asn211del, Leu212Ile, ins214Glu-Pro-Glu, Gly339Asp, Ser371Leu, Ser373Pro, Ser375Phe, Lys417Asn, Asn440Lys, Gly446Ser, Ser477Asn, Thr478Lys, Glu484Ala, Gln493Arg, Gly496Ser, Gln498Arg, Asn501Tyr, Tyr505His, Thr547Lys, Asp614Gly, His655Tyr, Asn679Lys, Pro681His, Asn764Lys, Asp796Tyr, Asn856Lys, Gln954His, Asn969Lys, Leu981Phe) (Arg682Ser, Arg685Ser, Lys986Pro, Val987Pro, R&D Systems). 2 × 10^6^ cryo-preserved PBMC samples were transferred in a 96-well U-bottom plate. Cells were first stained with Fc block (BD Biosciences) for 15 min at room temperature. Cells were then washed and stained with probe master mix containing 100 ng spike-A488, and 25 ng RBD-A647 for 1 h at 4C. Following incubation with antigen probes, cells were washed twice and stained with Blue Live Dead (Thermo Fischer) for 10 min at room temperature. Cells were washed again and stained with antibodies according to manufacturer protocols: BUV805-Mouse Anti-Human CD7, clone M-T701 BD Biosciences, BUV805-Mouse Anti-Human CD14, clone M5E2, BD Biosciences, BV711-Mouse Anti-Human CD19, clone HIB19, BD Biosciences, BUV395-Mouse Anti-Human CD20, clone 2H7, BD Biosciences, BUV737-Mouse Anti-Human CD21, clone B-ly4, BD Biosciences, BUV615-Mouse Anti-Human CD24, clone ML5, BD Biosciences, A700-Mouse Anti-Human CD27, clone L128, BD Biosciences, PE-CF594-Mouse Anti-Human CD38, clone HIT2, BD Biosciences, PecyPE-Cy-7-Mouse Anti-Human CD71, clone CY1G4, BD Biosciences, BV605-Mouse Anti-Human IgD, clone IA6-2, BD Biosciences, PerCP-Cy5.5 Mouse Anti-Human IgM, clone MHM-88, Biolegend, BV421-Mouse Anti-Human IgG, clone G18-145, BD Biosciences, APC-H7-Mouse Anti-Human HLA-DR, clone L243, Biolegend, and BV480-Rat Anti-Human CXCR5, clone RF8B2, BD Biosciences for 30 min on ice. Cells stained with the Spike Trimer were fixed with the transcription factor buffer (Thermo Fischer) and intra-cellularly stained for IRF4 (eFluor660, clone 3E4, Thermo Fischer) and Blimp-1 (PE-CF594, clone 6D3, BD Biosciences). Cells stained with RBD, and full Spike probes, were fixed overnight in 1% PFA. Samples were acquired on BD FACSymphony.

### Serology

A multiplexed bead-based flow cytometry assay referred to as microsphere affinity proteomics (MAP), was adapted for the detection of SARS-CoV-2 and the receptor-binding domain (RBD) antibodies as described.^24^, ^25^, ^26^, ^27^

### Measurement of neutralizing antibodies

Titrated amounts of sera were mixed with TCID100 of SARS-CoV-2 viruses (either Human 2019-nCOV strain 2019-mCoV/Italy-INM1, SARS-CoV-2/Norway/11421/2021 (Delta/B.1.617.2), or SARS-CoV-2/Norway/29450/2021 (Omicron/B.1.1.529) and tested for the ability to neutralize the infection of Vero E6 cells. After 4 days, SARS-CoV-2 infection of Vero E6 cells was measured as the expression of nucleocapsid detected by anti-SARS-CoV-2 nucleocapsid antibody (cat. 40,143-R004, Sino Biological), see Supplementary Methods for further details.

### Statistics

Comparative analyses of frequencies of cell subsets and marker expression are presented by GraphPad Prism version 9.3 with violin plots and dashed lines to indicate the median and interquartile range showing all data points, and the difference between the control and test group was tested using Mann–Whitney U test for unpaired data and Wilcoxon test on paired samples for the comparison between unstimulated and peptides stimulated samples. Tests were two-sided. Values of p < 0.05 were considered statistically significant. Correlations were calculated with Pearson's test. A correlation matrix was calculated comparing phenotypic and serological marker variables in a pairwise fashion, using the corr. test function from the psych CRAN package; the corrplot package was subsequently used to graphically display the correlation matrix. The resulting P values were adjusted for multiple testing using the Bonferroni method. Pearson's correlation coefficients were indicated by a heat scale whereby the red colour shows a positive linear correlation, and the blue colour shows a negative linear correlation. Spearman rank correlation was used when the normality assumptions for Pearson's correlation were not satisfied. The volcano plots and the correlation matrix were integrated as a package in CYTOGRAPHER®, ImmunoScape cloud-based analytical software.

### Role of the funding source

The funders of the study had no role in the study design, data collection, data analysis, data interpretation, writing of the report, or in the decision to submit the paper for publication.

## Results

### Patient IgG anti-RBD serological responses after vaccination and breakthrough infection (BTI)

Kidney transplant patients (KTR) were enrolled in an interventional trial to receive 3rd, 4th, or further doses of an mRNA vaccine [either BNT162b2 (Pfizer-BioNTech) or mRNA-1273 (Moderna)] against SARS-CoV-2. A repeat dose was administered if serological responses were less than 200 BAU/mL 1 month after the last delivery. At the start of this interventional trial, the cutoff was 10% of IgG anti-RBD responses in healthy donors; this corresponded to 317 patients that received the third dose (Supplementary Fig. S1a, Supplementary Table S1). The inclusion was amended to patients with <200 BAU/mL (using international WHO units), for dose 4. Fig. 1a shows the longitudinal follow-up of the serological response for dose 3 recipients (re-analysed to show patients with <200 BAU/mL) and intervention trial dose 4 recipients.

In addition to the interventional trial, non-included KTR patients were a few months later offered the 3rd dose and thereafter the 4th dose by the Norwegian Corona Vaccination program, regardless of the previous measurement of serological response (n = 3303). We included such KTR vaccine recipients in an observational trial, see Supplementary Fig. S1b for an overview. By comparison, healthy donors (HD) had significantly increased IgG anti-RBD responses at all time points, Supplementary Fig. S1c. The median IgG anti-RBD was 4890 BAU/mL [IQR, 1674–7585] in HD vs 2 BAU/mL [IQR, 1.9–3.7] in KTR (p < 0.0001) 1 mo post dose 2 (D2m1), Fig. 1b. Thus, 406/432 (94%) KTR were non-responders (<5 BAU/mL, 79%) or low responders (<200 BAU/mL, 15%), Fig. 1c. One mo after dose 3, the median had increased to 25.9 BAU/mL [IQR, 2.4–492] in KTR vs 11,731 BAU/mL [IQR, 5938–22292] in HD, Fig. 1b. Thus, 68% were still non-responders or low responders (361/857; 220/857); and 32% were responders or good responders (157/857; 119/857). One mo after the fourth dose, 54% were either responders (320/1612) or good responders (542/1612), while 46% were non-responders (516/1612) or low responders (234/1612). The analysis of the 5th vaccination in KTR revealed a significantly higher anti-RBD IgG titer (median values from 479 [IQR, 3.5–4290] at D4m1 to 2444 BAU/mL [IQR, 8–14515] at D5m1, n = 406, p < 0.0001). So, one month after dose 5, 66% of KTR were responders (54/406), or good responders (212/406). Only 37 patients were analysed for dose 6 responses, but also here 62% were responders (9/37) or good responders (14/37). In comparison, nearly all HD were responders or good responders already 1 m after dose 2 (167/173, 96%), and nearly all were good responders 1 mo after dose 3 (182/184, 98%).

We next turned to non-responders after 4th dose and focused on paired responses after dose 5. 132/326 (40%) were non-responders at D4m1. After dose 5, 43/132 (32%) had seroconverted, including 21% responders (15/132) or good responders (9/132). These included 25% of preserved serological responses (D5m1/D4m1 BAU/mL ratio = 0.98, n = 326) and 75% of improvement or seroconversion of previous non-responders (75% percentile of D5m1/D4m1 BAU/mL ratio = 11.88, n = 326). Thus, even for patients that were refractory to the first four doses, further vaccination allowed responses in a third of the patients. Similar conversions were found after dose 6, although 14/37 (38%) remained non-responders. IgG anti-RBD decayed a median of 1.5-fold (IQR 0.8–5.4) between D4m1 and 5th vaccination (n = 60) and by a 3.6-fold decrease (IQR 1.6–7.8) if only KTR with vaccine-elicited seroconversion were included (n = 38) (Supplementary Fig. S1d).

We next assessed the effect of BTI in the KTR interventional cohort and HD (the majority had received 3rd dose), the individuals had been sampled before and after they presented positive PCR tests for SARS-CoV-2 (n = 32 and n = 12 for KTR and HD respectively). KTR patients with BTI developed significantly increased (p < 0.0001) IgG anti-RBD and 28 of 32 (87.5%) were above 2000 BAU/mL after BTI. These high-responder patients had lower anti-RBD levels as compared to HD before (p = 0.0002) and after BTI (p = 0.0014), Fig. 1d.

### Development of severe COVID-19 in vaccinated KTR–Efficacy of vaccination to prevent hospitalization and death

The efficacy of vaccination in the observational cohort was followed for patients that developed COVID-19, Fig. 1e.

KTR patients had COVID-19 mortality of 23% before vaccination.^1^^,^^28^^,^^29^ The Norwegian KTR population had a relatively low incidence of COVID-19 during the Wuhan/Alpha/Delta waves but a dramatic increase during Omicron VOC (variant of concern) from December 2021. During the initial waves, the Norwegian government imposed several COVID-19-related restrictions, during which KTR tended to be extremely cautious.^30^ Following releases in restrictions, a total of 1098 (30%) KTR developed COVID-19 from December 2021 through October 2022 with a reported mortality of 3.7% (41 KTR treated in the ICU), and with a reported hospitalization rate of 23% (251 KTR). The incidence was highest from February 2022 corresponding with the initial Omicron BA.1 wave. Fig. 1e shows a Kaplan Meier analysis of patient survival by COVID severity during the entire pandemic (through October 2022) with graft- and death-censored graft survival in Supplementary Fig. S1d–f. See also our recently published multistate competing risk analysis that showed that seroconversion in KTR prevented fatal disease progression and reduced the risk of COVID-19 death.^31^

### KTR patient overview

**Fig. 2:**
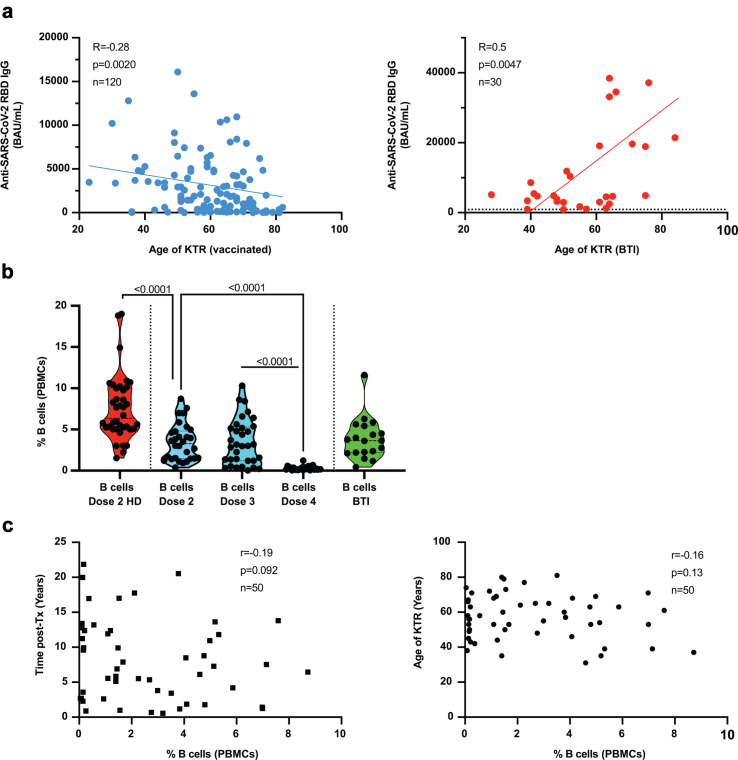
**KTR time after transplantation and quantification of B cells in the interventional cohort**. a. Anti-SARS-CoV-2 RBD IgG (BAU/mL) correlation to age in vaccinated KTR (dose 2–4, left), or in KTR after BTI (right). A linear regression is shown and Spearman's test was used for correlation analysis. b. Percentage of B cells in PBMC of healthy donors after 2 doses, and KTR after doses 2 to 4 or after BTI. c. Percentage of B cells in peripheral blood of KTR and correlation to the time after kidney transplantation and to the age of patients at enrolment in the vaccine study. Pearson's test was used for correlation analysis.

PBMC of KTR from the intervention cohort were cryo-preserved to allow in-depth analyses. These patients were recruited from 223 KTRs that lived close to the hospital, Supplementary Table S1 details the demographic and clinical parameters of the patients. The KTR patients had a median age of 58 y [IQR, 44–68] (doses 2 and 3 or dose 4) and had a median time after transplantation of 7 y [IQR, 4–13] and 8 y [IQR, 2.5–13] respectively. The age and time post-transplantation were not significantly different between doses 2, 3, and 4 recipients. Serological responses inversely correlated with age (Fig. 2a, p = 0.0020), except in BTI where responses increased with age (Fig. 2a, p = 0.0005).

### KTR and reduced B cell levels

Next, we tried to decipher the immune basis of the low responsiveness to vaccines in KTR. First, we assessed the frequency of total B cells to determine if immune suppressive therapy had impeded humoral immunity. PBMC from KTR had significantly reduced B cell frequencies, with a median of 3.3% [IQR, 1.5–4.8] and 3.0% [IQR, 1.2–5.3] in dose 2 and dose 3 recipients respectively (p < 0.0001 for both) compared with 6.4% [IQR, 5.1–10] in HD, Fig. 2b. The selection of serological non-responders (<200 BAU/mL) after 3 doses of vaccines for the 4th dose intervention resulted in patients with very low B cell frequency of 0.17% [IQR, 0.12–0.31]. The frequency of B cells did not correlate with age nor time post-transplantation, Fig. 2c. Interestingly, KTR patients that developed BTI had a median B cell frequency of 3.6% [IQR, 2.2–5.4], Fig. 2b. The cause of the relative paucity of B cells in some KTR remains unclear, but the finding required further analyses, especially of Spike-specific B cell responses.

### Spike-binding B cells in KTR

**Fig. 3:**
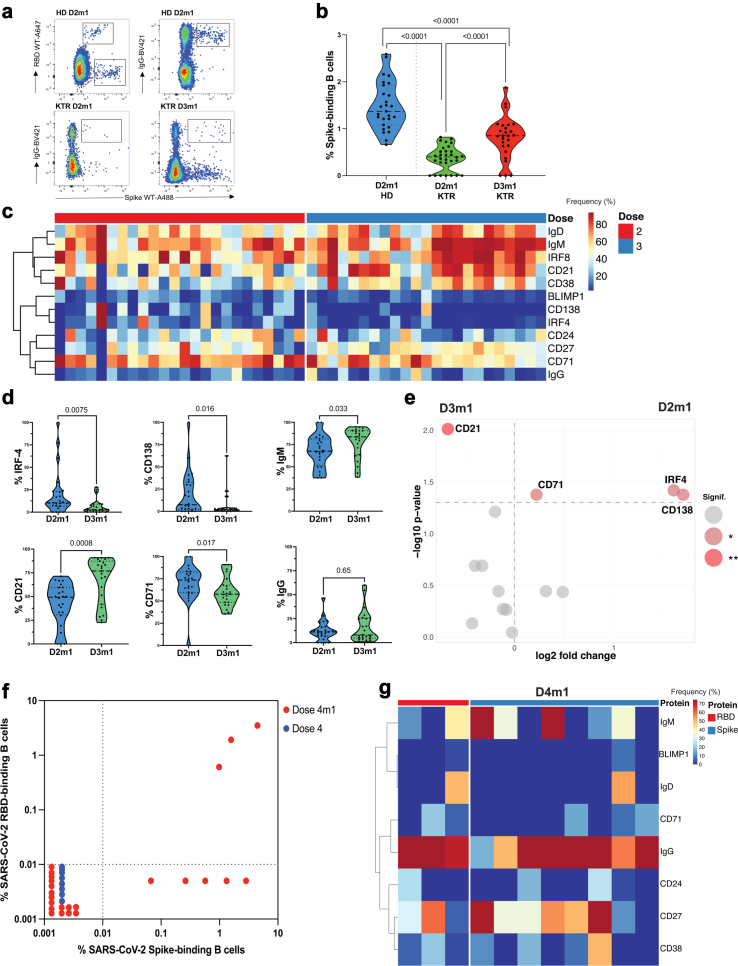
**RBD and Spike-binding B cells during vaccination in KTR**. a. Detection of Spike-binding B cells in PBMC of KTR 1mo after dose 2 (D2m1), 1mo after dose 3 (D3m1) and in HD (D2m1). IgG vs Spike (WT) in gated CD19^+^ B cells is shown. b. The frequency of Spike-binding B cells in healthy donors (blue, n = 27) and KTR one month after dose 2 (green, n = 30) and dose 3 (red, n = 26). c. Phenotype of Spike-binding B cells. Heat plots show the phenotype of Spike- or RBD-binding B cells in KTR 1mo after dose 2 (n = 24) or 3 (n = 23). The frequency of each marker is displayed and the automatic hierarchical clustering of Spike-binding B cells for each marker is shown. d. Percent of Spike-binding B cells in KTR that express IgM, IgD, IRF-4, CD71, CD138, or IgG at D2m1 (n = 24) and D3m1 (n = 23) is shown. e. Longitudinal signature of Spike-binding B cells in KTR. The phenotype of Spike-binding B cells was compared between dose 2 (n = 24) and dose 3 (n = 23) and significant differences were displayed in the volcano plot. Only markers with p < 0.05 (light red) and p < 0.01 (red) are annotated on the graph. f. Quantification of Spike-binding and RBD-binding B cells after dose 4. The frequency of RBD-vs Spike-binding B cells in total live B cells is represented for KTR before (n = 8) and 1mo after dose 4 (n = 24). g. Phenotype of Spike-binding and RBD-binding B cells in responder patients after dose 4 (n = 8). A heat plot shows the phenotype of Spike-specific and RBD-specific B cells in KTR one month after dose 4 of the mRNA vaccine. The frequency of each marker is displayed and automatic hierarchical clustering of Spike-binding B cells for each marker is shown.

Long-lasting humoral immunity requires the persistence of antigen-specific memory B cells and isotype switching. The combination of anti-Spike (full protein) and anti-RBD tetramer staining with deep immunophenotyping enabled us to address the question of whether the reduced serological response in KTR was accompanied by decreased frequency of SARS-CoV-2 Spike-binding B cells after vaccination. Few Spike-binding B cells were detectable in comparison to HD and their phenotype was not associated with memory IgG^+^, Fig. 3a. The median Spike-binding B cells frequency after 2nd or 3rd dose in KTR was only 0.4% [IQR, 0.2–0.54] (n = 30) and 0.85% [IQR, 0.6–1.1] (n = 26) respectively compared to 1.4% [IQR, 1.1–1.9] in healthy controls (n = 27), Fig. 3b. Moreover, the phenotype of these B cells was mostly non-switched IgM/IgD CD21^+^CD27^+/−^CD71^+/−^ CD38^+/−^ B cells with few memory IgG^+^ antibody-secreting cells and almost no plasma blast defined as CD38^Hi^BLIMP-1^+^ IRF-4^+^CD138^+^, Fig. 3c, see^32^ for B cell subsets. The modulation of individual markers expressed on Spike binding B cells in KTR 1 mo after doses 2 and 3 is shown in Fig. 3d (IgM^+^, IgG^+^, CD21^+^, IRF-1^+^, CD71^+^, or CD138^+^).

In contrast, RBD or Spike binding B cells in HD were mostly IgG^+^ CD27^+^ CD24^−^CD38^−^CD71^-^ memory B cells (up to 6 months after vaccination) and persisted despite the relatively decreased frequency of memory B cells over time as reported by others,^33^ Supplementary Fig. S2a–c.

We next performed an unsupervised phenotypic analysis of Spike-binding B cells, demonstrating five distinct clusters (by Phenograph, Supplementary Fig. S2d**)**. The distribution of B cell clusters changed over the doses of vaccines. The response after dose 2 was enriched for IgG^low^ plasma cells (cluster 1 (c1), BLIMP-1^+^CD138^+^IRF-4^+^), IgM^+^IgD^−^ memory, and B reg-like B cells^32^ (CD24^+^CD27^+^, c2), IgG^+^ early memory B cells (CD20^+^CD71^+^CD24^Low^HLA-DR^+^, c4) and IgD^+^ Naive B cells (CD20^+^ CD21^+^CD24^Low^, c5), Supplementary Fig. S2e and f. After dose 3, we found a further surge of non-switched B cells, 45% were (IgM/D^+^HLA-DR^low^CD20^+/low^CD38^Hi^IRF8^+^BLIMP-1^−^CD138^−^IRF-4^−^) indicating that two doses of vaccination had not yet developed sufficient memory cells and that de novo activation was still ongoing in KTR even after dose 3.

The statistical analysis was represented by a volcano plot to illustrate and quantify the specific markers expressed after the different doses of vaccine in KTR, Fig. 3h. It showed that dose 2 Spike-binding B cells significantly expressed plasmablast markers (CD138, IRF4) as well as the early activation marker of memory B cell (CD71)^32^ while KTR after dose 3 had significantly increased CD21 expression (such as in c3 CD21^+^CD27^+^CD38^+^ activated memory B cells), Fig. 3e.

Since the serological responses improved by dose 4, we next investigated if further vaccination also rectified the deficit of mature B cells. However, in these patients selected for lack of serological response that had few B cells (see above) only a minority of 12.5% KTR had detectable RBD and Spike-binding B cells after the 4th dose of the vaccine (3/24). Five of 24 (21%) had Spike-binding but not RBD-binding B cells, Fig. 3f. Thus, 16/24 (66%) had no detectable Spike/RBD-binding B cells, Fig. 3f. In contrast, most of HD (87.5%, n = 16) had Spike/RBD-binding B cells already after dose 2 (Supplementary Fig. S2). Nevertheless, the phenotype of Spike and RBD binding B cells was enriched for IgG^+^ B cells, suggesting the gradual emergence of mature B cell responses after the fourth dose. Specific B cells were mostly CD27^+^IgG^+^CD24^−^CD21^−^CD38^−/low^CD71^-^ switched memory B cells, Fig. 3g, a B cell subtype that is likely to rapidly respond to BTI.

### BTI and development of cross-reactive B cells in KTR

**Fig. 4:**
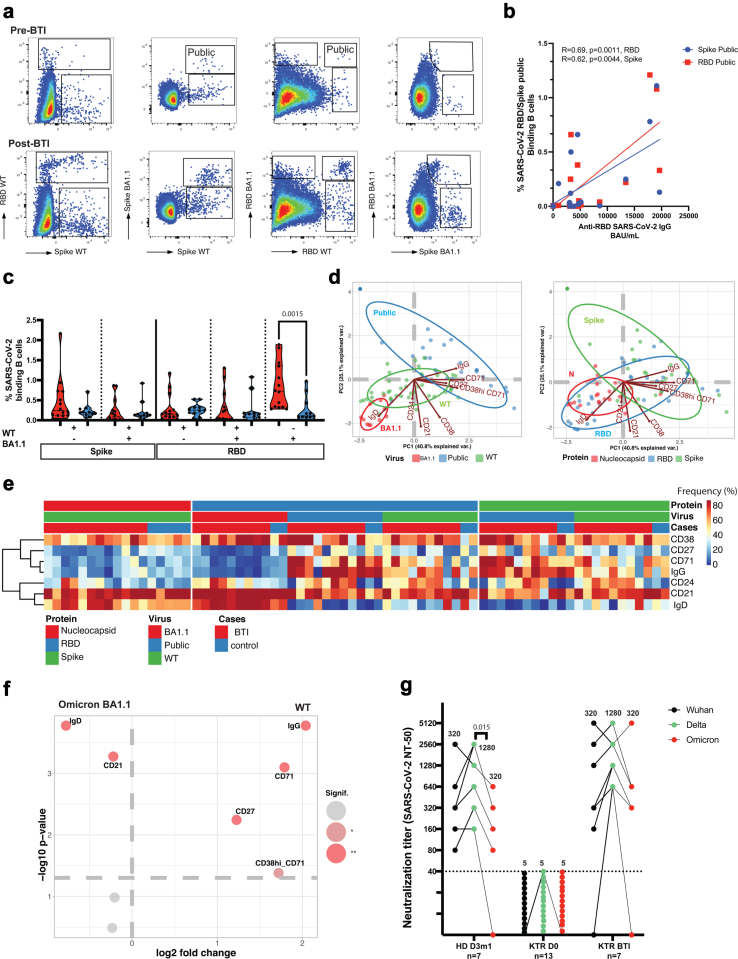
**Humoral and B cell immunity during Omicron and Delta breakthrough infection**. a. Identification of Spike and RBD-binding B cells before (top) and after BTI (bottom). As indicated, representative dot plots show gated, live CD19^+^ B cells that bind RBD and/or Spike. Cross-reactive B cells that bind both WT and BA.1.1 are shown in the region marked Public, for Spike and RBD. Other regions show Spike-only, or RBD-only binding B cells as indicated. b. Pearson correlation between serological and B cell responses in KTR after BTI infection. IgG anti-SARS-CoV-2 RBD IgG vs the frequency of Spike public- or RBD public-binding B cells (blue and red, respectively, n = 25). c. Quantification of Spike-binding and RBD-binding B cells during BTI in KTR (red, n = 14) and HD (blue, n = 11). The frequency of Nucleocapsid, wild-type, and/or mutated RBD- and Spike-binding B cells in total live B cells 1mo after BTI. Two-tailed Wilcoxon paired test, p < 0.01. d. Distribution of WT and BA1.1 Spike-binding and RBD-binding B cells after Omicron BTI in KTR. Visualization by principal component analysis (PCA) biplots of selected markers as indicated (arrows) and location of WT and/or VOC (left); or Nucleocapsid vs RBD vs Spike binding B cells (right), red, blue, and green as indicated. The origin of epitopes and each epitope are visualized by a colour code and the distribution is summarized by an automatically generated ellipse. e. Characterization of SARS-CoV-2-binding B cells. A heat plot shows the phenotype of Nucleocapsid, RBD, and Spike-specific B cells in KTR and HD after BTI (n = 12; n = 6). The frequency of each marker is displayed, and the automatic hierarchical clustering of markers expressed by SARS-CoV-2 binding B cells for each patient is shown. f. Specific signature of RBD-binding B cells during BTI in KTR (n = 12). The phenotype of RBD-binding B cells was compared between cells able to bind exclusively wild-type or mutated RBD. The significant differences are displayed in the volcano plot. Only markers with p < 0.05 (light red) and p < 0.01 (red) are annotated. g. Neutralizing antibody titers in plasma samples of COVID-19 vaccinated and KTR after BTI. Plasma samples were collected after the 3rd dose in healthy donors (n = 7), from non-vaccinated kidney transplanted patients (n = 13), and from vaccinated infected KTR (n = 7). The dotted line indicates the cut-off of the assay (first serum dilution was 1:40). The samples that did not show any inhibition of viral infection are presented as 5 for plotting purposes. The median values of the neutralization titers are indicated in the graph. Statistical analysis was performed using the Friedman test with Dunn's multiple comparisons tests.

Despite the hypo responsiveness of KTR B cells to vaccines, natural infection by the circulating strain of SARS-CoV-2 (Omicron BA.1) was able to induce seroconversion in KTR as shown above, Fig. 1d. An important question was if this increase was related to boosted vaccine immunity, or de novo immunity not related to vaccination. To investigate the impact of vaccines, we, therefore, compared B cell immunity in the KTR before and after BTI. We focused on B cell's ability to recognize and bind Wild Type (WT) Spike, as found in the vaccine, as well as Omicron BA1.1 Spike as most KTR developed BTI with the Omicron VOC, Fig. 4, and Supplementary Fig. S3a. Before the BTI (pre-BTI), KTR had few B cells that bound WT RBD and Spike (derived from the vaccine), Fig. 4a, and Supplementary Fig. S3b. About 30% (vs 41% in HD with BTI) of the Spike binding B cells were cross-reactive and could also bind Spike BA.1.1 (Omicron), on a shared, public epitope, Fig. 4a and b (region marked “Public”). Similarly, we found cross-reactive B cells that could bind a public epitope on both WT and BA1.1 RBD. In addition, some B cells bound only to RBD WT. However, very few B cells bound only to RBD BA1.1 and not the RBD WT. Moreover, the serological response during BTI was a good indicator of the development of B cell responses. The frequency of the cross-reactive Spike- and RBD-binding B cells correlated positively with IgG anti-RBD (BAU/mL) levels, Fig. 4b. This indicated that the vaccine-induced B cells of the cross-reactive type were further expanded by the Omicron infection and that this response accounted for (correlated with) the seroconversion.

In fact, after BTI, Spike- and RBD binding B cells expanded to levels similar to or higher than that found after BTI in HD, Fig. 4c. This included WT as well as public Spike and RBD. The frequency of Omicron BA1.1-only RBD binding B cells (that can recognize Omicron VOC, but not WT RBD) was also significantly increased, Fig. 4c. Next, we compared the anti-Spike humoral response through phenotyping analysis. The emergence of WT and cross-reactive B cells in BTI was associated with IgG and B cell early activation markers on memory B cells (CD27, CD38, CD71, Fig. 4d–e, and Supplementary Fig. S3c–e). This demonstrated a vaccine imprint and fully matured B cell response after BTI. In comparison, de novo Omicron BA1.1-only RBD-responses were IgD^+^ (Fig. 4d). A similar newly primed IgD phenotype was found for emergent Nucleocapsid-binding B cells (IgD^+^CD21^+^CD38^+^CD27^+/−^CD24^−/+^), Fig. 4d–e. Statistical analysis of B cell phenotypes in volcano plots showed significantly increased IgD and CD21 in Omicron BA.1-only binding B cells, while WT-binding B cells significantly expressed IgG, CD27 (memory) CD38 (activation), and CD71 (early activation marker of memory B cells), and CD38^Hi^CD71^+^ (plasmablasts), Fig. 4f. The data, therefore, showed a difference of maturity between i) vaccine related B cell responses further expanded in BTI that had fully matured, and ii) newly primed Omicron-only Spike and Nucleocapsid B cell responses.

Finally, to formally demonstrate the full functionality of this vaccine B cell immunity expanded by BTI, we tested if sera could neutralize VOC and found that post-BTI KTR sera neutralized broadly, demonstrating cross-reactivity with WT (Wuhan), Delta, as well as BA1.1 (Omicron) VOC, Fig. 4g, and Supplementary Fig. S3f. Results suggested that vaccination in KTR induced heterogeneous humoral responses that could be boosted by natural viral infection, including VOC.

### KTR and development of spike-specific CD8 T cells

**Fig. 5:**
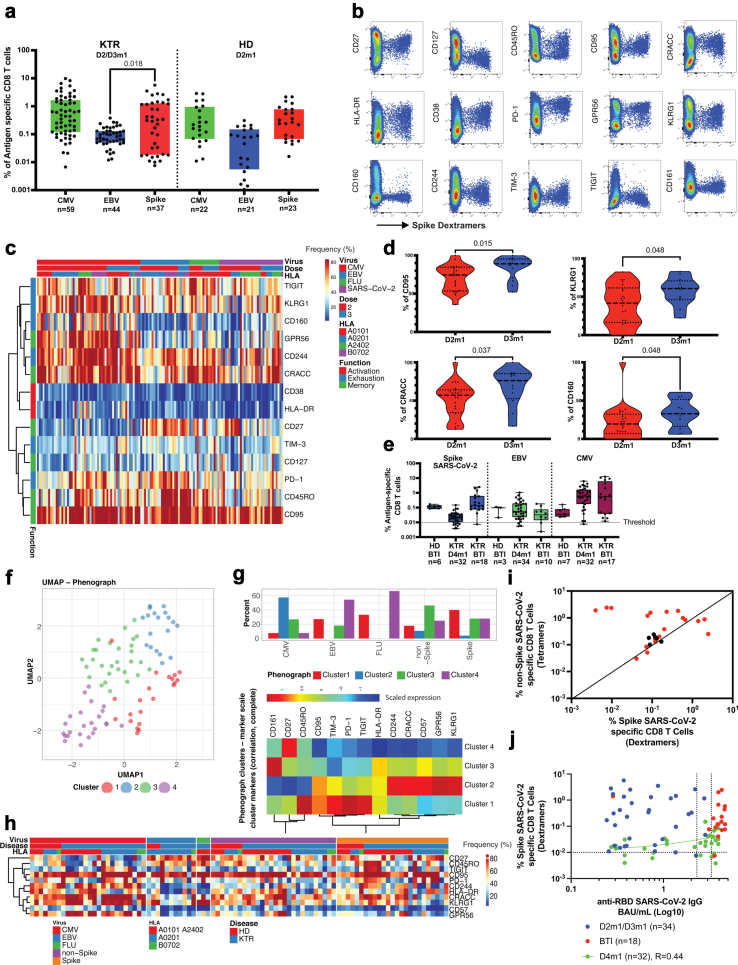
**Cytotoxic cellular immunity during vaccination and BTI in KTR**. a. Quantifying SARS-CoV-2 Spike-specific CD8 T cells defined by peptide: HLA-multimers in vaccinated KTR. b. Ex vivo immune phenotype of SARS-CoV-2 Spike-specific CD8 T cells. Representative dot plots of live CD8 T cells from HLA∗A0201 restricted patients. SARS-CoV-2 Spike-specific CD8 T cells are identified along X-axis and combined with phenotypic markers on Y-axis. c. Characterization of SARS-CoV-2 Spike-specific CD8 T cells. A cold-to-hot heatmap represents the scaled frequency of each marker expressed by antigen-specific CD8 T cells. The frequency of each marker is displayed, and the automatic hierarchical clustering of markers expressed by HLA-class I restricted multimers and of patients is shown. The top three rows indicate the epitope-derived viruses, the dose of vaccines, and HLA for A and B alleles. d. Longitudinal characterization of SARS-CoV-2 Spike-specific CD8 T cells in Kidney Tx patients. The phenotype of SARS-CoV-2 Spike-specific CD8 T cells was compared one month after the second (n = 22) or third (n = 12) dose of the vaccine. Mann–Whitney U test was used to compare the median frequencies of markers. e. Quantification of SARS-CoV-2 Spike specific CD8 T cells defined by multimers in KTR in response to fourth dose vaccine or breakthrough infection. f. Visualization by an UMAP-plot of selected markers (described in c.) in virus-specific CD8 T cells. The clusters were automatically identified by phenograph software (left). Clusters distribution of virus-specific CD8 T cells during BTI in KTR. The frequency of clusters during the month after the diagnosis of the BTI was identified by phenograph (middle). g. Characterization of clusters from antigen-specific CD8 T cells during BTI in KTR. The normalized frequency of each marker is visualized by a cold-to-hot heat map and an automatic hierarchical clustering of virus-specific CD8 T cells for each marker is shown. h. Characterization of SARS-CoV-2- Spike- and non-Spike-specific CD8 T cells after BTI in KTR. A heat plot shows the phenotype of Nucleocapsid, RBD, and Spike-specific B cells in KTR and HD after BTI. The frequency of each marker is displayed, and the automatic hierarchical clustering of markers expressed by SARS-CoV-2-specific or virus-control CD8 T cells for each patient is shown. The top three rows indicate the epitope-derived viruses, the immune status of donors (HD vs KTR), and HLA for A and B alleles. i. Biplot showing ex vivo detection of MHC class I restricted multimers CD8 T cells directed against epitopes derived from Spike and non-Spike (Nucleocapsid, ORFs) in BTI KTR. Red and black circles represented T cells from BTI in KTR (n = 20) and HD (n = 6) respectively. j. Biplot showing anti-SARS-CoV-2 humoral and cytotoxic immune response in KTR. ex vivo detection of MHC class I restricted multimers CD8 T cells directed against epitopes derived from Spike and serological measurement of IgG anti-SARS-CoV-2 RBD (BAU/mL) in KTR one month after the second/third (blue) or fourth dose of vaccine (green) from interventional cohort or after BTI. The correlation was significant only for KTR after the 4th dose (Spearman, R = 0.44, p = 0.011, n = 32). Dashed lines represent indicative values of cellular and humoral responsiveness threshold.

To quantify vaccine-induced T cell immunity, we next analysed CD8 T cells specific for Spike peptides using HLA class I-restricted multimers (in patients or controls that expressed the following alleles: HLA∗A0101, HLA∗A0201, HLA∗A2402, HLA∗B0702). Cells were also stained with CMV- and EBV-multimers as internal controls (See Methods and Supplementary Table S2). We detected Spike-vaccine-specific CD8 T cells in KTR, and the median frequency (0.31%, [IQR, 0.0185–1.27]) was increased and significantly different from the frequency of EBV-specific T cells (0.086%, [IQR, 0.05–0.13], p = 0.018). However, the 25% percentile of the Spike specific T cell frequencies in KTR patients was three-fold lower compared to vaccinated healthy donors (0.0185%, n = 37 vs 0.068%, n = 23), suggesting incomplete development of T cell responses in these patients, which could be only partially explained by HLA distribution, Fig. 5a and Supplementary Fig. 4a. The ex-vivo frequency of Spike-specific CD8 T cells remained stable after the third dose of the vaccine in KTR, Supplementary Fig. S4b.

We next performed immune-phenotyping of T cells to identify markers associated with protective responses and mild SARS-CoV-2 infection. A vaccinated healthy donor with a successful generation of Spike-peptide: HLA-specific T cells is shown in Fig. 5b, with dot plots showing SARS-CoV-2 Spike-specific-dextramer staining vs phenotypical markers on gated live CD8 T cells. The ex-vivo phenotype of SARS-CoV-2 Spike-specific CD8 T cells differed from CMV-, EBV-, or FLU-specific CD8 T cells, Supplementary Fig. 5c and d. Further analysis is presented with hierarchical clustering for markers in Fig. 5c for KTR and in Supplementary Fig. 4d and e for HD. Most Spike-peptide: HLA-specific T cells were memory (CD27, CD45RO, CD95), recently activated (CD38, PD-1, HLA-DR, combined with the down-modulation of CD127), had acquired expression of effector markers (CRACC, KLRG1, GPR56) and did not express exhaustion or senescence markers (CD160, CD244, TIM-3, TIGIT, CD161).

We sought to determine if the booster dose (Dose 3) can modify the phenotype of previously vaccine-generated Spike-specific CD8 T cells. We detected a significant maturation in the expression of differentiation markers (CD95) associated with the acquisition of effector function markers (CRACC, KLRG1) and exhaustion (CD160) from 4 weeks after vaccine dose 2–4 weeks after dose 3, demonstrating restimulation of pre-existing antigen-specific T cells and therefore, consolidated effector responses, Fig. 5d.

### Analysis of spike-specific CD8 T cells in KTR after dose 4 and in BTI

The KTR Patients that had low serological responses to the 3rd dose received the 4th dose in the interventional trial. After 4 weeks the median frequency of Spike-specific T cells was 0.022%, [IQR, 0.011–0.038] (n = 32), more than 10-fold less than that measured in KTR after dose 2/3 (p < 0.0001), Fig. 5 e. Moreover, 25% of Spike-specific CD8 T cells (8/32) had a very low frequency, below the 0.01% threshold.

In contrast to the interventional KTR cohort, patients after BTI demonstrated the same frequency of Spike-specific T cells as seen in HD, Fig. 5 e, with a median frequency of 0.135% [IQR, 0.08–0.6] Spike-specific dextramers (p < 0.0001, n = 18). PBMCs of BTI patients were also stained with peptide multimers to detect non-spike specific T cells (M, N, and O, see Methods). Unbiased phenotypic analyses identified 4 clusters of CD8 T cells specific for CMV, EBV, Flu, Spike, and non-Spike, Fig. 5f–g, and Supplementary Fig. 4f and g. CMV-specific T cells were represented in cluster 2, defined by a signature of late-differentiated T cells (KLRG1^Hi^GPR56^Hi^CRACC^Hi^CD244^Hi^), with a strong correlation between these markers (Supplementary Fig. S4g). EBV- and Flu-specific CD8 T cells were dominated by cluster 4 as resting early memory T cells (CD27^+^) and by cluster 1 characterized by memory markers (CD95 and CD45RO) and inhibitory receptors expression (TIGIT^+^TIM-3^+^PD-1^+^). C1 was dominant for Spike-specific responses suggesting the specificity of this cluster for acute immune reaction (viral infection and vaccination). Non-Spike-specific CD8 T cells were mainly located in cluster 3, which identified recently activated T cells (HLA-DR^+^). The frequency of individual markers is represented in Fig. 5h for each virus-specific Class I restricted multimers based on HLA specificity, viruses, and disease status.

To evaluate whether the cellular response was similarly induced against Spike and non-Spike derived epitopes, both responses were compared in the same individuals post-BTI. We plotted the frequency of Spike- and non-spike-specific T cells after BTI in KTR and HD in a biplot, Fig. 5i. While most individuals were found on the diagonal (similar frequencies of Spike vs non-spike frequencies as observed for BTI in HD represented with black dots), 6/19 (31%) of KTR had non-spike dominant T cells (>10–100 fold) suggesting less successful vaccine (or at least Spike) immunity. Next, we tried to establish if humoral and cellular immunity was associated with our KTR cohort. The magnitude of anti-Spike humoral and cytotoxic T cells was not correlated during the 2nd or 3rd dose in KTR and after BTI but was positively associated in the interventional cohort after the 4th dose (p = 0.037, r = 0.37, n = 32), Fig. 5j.

### Analysis of in vitro responses in KTR, CD4, and CD8 T cells

**Fig. 6:**
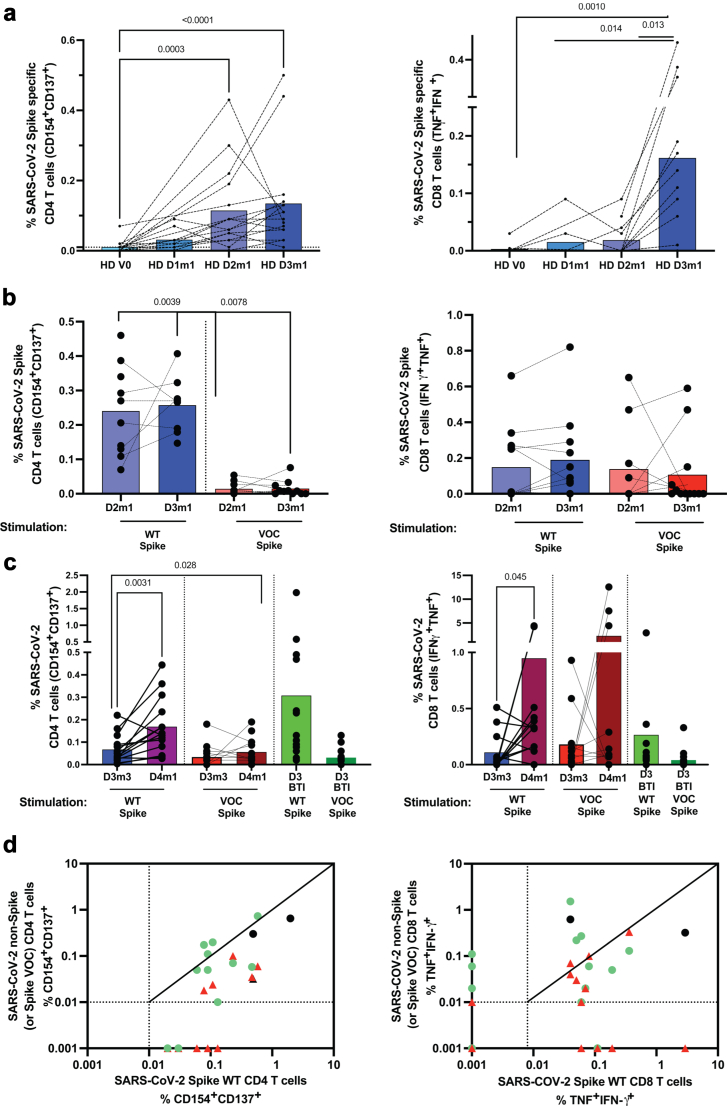
**In vitro T cell responses to Spike and non-spike peptides**. a. Longitudinal follow-up of functional responses of SARS-CoV-2 specific T cells in healthy donors before vaccination and one month after dose 1, dose 2, and dose 3 (n = 16). Spike-specific CD4 T cells responded with up-regulation of CD154 and CD137, left. Spike-specific CD8 T cells responded by the secretion of TNF and/or IFN-γ, right. b. Longitudinal follow-up of functional responses of SARS-CoV-2 specific T cells in KTR one month after dose 2 or 3 (n = 10). Spike-specific T cells were identified as in a. Only responder patients (CD4, left, CD8, right) are shown. c. Longitudinal follow-up of functional responses of SARS-CoV-2 specific T cells in KTR one month after dose 4 (n = 14) or BTI (n = 15). Spike-specific T cells were identified as in b. Only responder patients (CD4, left, and CD8, right) are shown. d. Biplot showing in vitro responses of T cells to non-spike peptide pools vs spike-peptides or mutated Spike in KTR (n = 14). Green and black circles represented T cells directed against Spike and non-Spike epitopes from omicron and Delta-infected patients respectively. The response directed against the specific Omicron mutated epitopes is represented by red triangles (n = 13).

The other component of the vaccine-elicited cellular immune response is constituted by the helper response, including the functional cooperation with B and CD8 T cells. We next turned to functional responses of CD4 and CD8 T cells after stimulation with Spike peptides in HD and KTR. All HD controls had detectable CD4 and CD8 T cell responses after the third dose of vaccine, Fig. 6a and Supplementary Fig. S5a. In contrast, only 8/12 (67%) of KTR had CD4 T cell responses to Spike peptides (CD154^+^CD137^+^) after dose 3, while only 7/12 (58%) of KTR had developed Spike-specific CD8 T cells, Fig. 6b (only KTR with positive responses are shown) and Supplementary Fig. S5b. Patients with no seroconversion were tested before and after the administration of the 4th dose. More than 75% of KTR were without T cell responses (14/18) three months after dose 3. However, the vaccine-elicited T cell immunity increased to 70% and 33% for CD4 and CD8 T cells respectively after the 4th dose. We found a significantly improved responsiveness among dose 4 responders of both CD4 and CD8 T cells, (p = 0.0031, n = 14, and p = 0.049, n = 12 respectively), Fig. 6c.

### Analysis of in vitro responses in KTR after BTI

To evaluate if cellular immunity was robust enough to limit the severe form of the disease after BTI (including systemic activation) and to sustain humoral immunity, we analysed immune responses towards spike and non-Spike peptides after BTI. Most KTR after BTI in dose 3 recipients (15/16) had detectable Spike-specific responses (significantly increased compared to after dose 2) in CD4 T cells. We found significantly increased CD4 T cell responses in KTR patients from Dose 3 (3 mo) to BTI (p = 0.028, n = 15), Fig. 6c. 10/13 BTI patients had all also developed specific CD4 and CD8 T cell responses towards non-Spike peptides (MNO) and including two patients with no response for CD4 and CD8 T cells, Fig. 6d and Supplementary Fig. S5c and d. All BTI patients had Spike-specific CD4 T cell responses, but 3 lacked detectable CD8 responses. T cell responses to Omicron-specific Spike peptides were variable but limited in comparison to response against WT Spike. The responses to Spike VOC were not detected in 3 and 5 KTR BTI patients for CD4 and CD8 T cell responses respectively. Further investigations are needed to evaluate if hybrid immunity in KTR, with incomplete T cell response, will be sufficient to limit the consequences of viral infection by new variants such as BA.5, XBB.1, or BQ.1 (or other derivatives).

### Integrative analysis of responses after 4th dose

The main goal of this study is to document the vaccine and hybrid immunity in immune-compromised patients. We next compared the overall immune responses in all dimensions, from functional CD4^,^ and CD8 T cell responses, to serological responses after four vaccinations (in serological non-responder after three vaccine doses). Cellular and humoral responses were defined as positive with respective 0.01% of Spike-specific T cells in vitro after restimulation with overlapping peptides covering Spike and 2000 BAU/mL of anti-RBD IgG as thresholds, Supplementary Fig. S6. We found that serology and T-cell responses were dissociated. Only 7% (4/58) of KTRs had a complete immune response, defined as triple responders for anti-Spike CD4, CD8 T cells, and antibodies. Double responders were detected in 9% (5/58) for combined Abs/CD8, 14% (8/58) for combined Abs/CD4, and 21% (12/58) for combined CD4/CD8 immune responses. Patients with only one arm of vaccine-elicited immunity were represented at 17% (10/58) for the humoral response, 24% (14/58) for the cytotoxic T-cell response, and 45% (26/58) for the helper T-cell response. Global hypo-responsiveness to the vaccine (no anti-RBD IgG, no functional T cells, no detectable Spike-specific CD8 T cells ex-vivo) of 15% of KTR (3/20) raised concerns about their susceptibilities to severe form of COVID-19 in a future wave of VOC infection.

### Inflammatory status before vaccination and after BTI in KTR

Finally, we investigated plasma to decipher the inflammation in non-vaccinated KTR and in KTR after BTI. A pre-vaccine screen of inflammatory plasma markers in KTR showed a significant increase in oxidative stress marker (GDF-15) and innate immune markers such as MPO, and Calprotectin (associated with neutrophil activation), which also positively correlated with IL-6 concentration, Supplementary Fig. S7a and b.

Systemic inflammatory markers were limited after BTI in HD, as also described before.^23^ In contrast, we found that KTR after BTI developed significant inflammation where Calprotectin, MPO, GDF-15, LBP, and D-Dimer concentrations were the most significantly increased (p < 0.0001), Supplementary Fig. S7 c and d. This was accompanied by significantly upregulated markers of systemic inflammation (IL-6, p = 0.027 and IP-10, p = 0.0002), cytokines that also support B cell and plasma cell differentiation, platelet activation (PF4, p = 0.0003), and monocyte activation (sCD14, p = 0.0045, sCD163, p = 0.008, and Galectin-9, p = 0.010) post-BTI in KTR compared to post-BTI in HD. We also found evidence of proinflammatory cytokine sets that correlated in KTR patients such as IL-18, IL-6, and IP-10 (pro-inflammatory cytokines that facilitate type 1 responses and B cell differentiation) Supplementary Fig. S7e.

## Discussion

We found that KTR had a reduced serological as well as T and B cell response to SARS-CoV-2 vaccination. Nevertheless, two-thirds of our patients developed relatively normal CD8 responses in terms of frequency of T cells with TCR that bound Spike-specific Dextramers and phenotype after 3 vaccine doses in comparison to vaccinated HD. This was matched by CD4 and CD8 T cell responses in vitro to the SARS-CoV-2 peptide. This cellular response was associated with a vaccine-elicited humoral response. We detected Spike- and RBD-binding B cells after 2nd and 3rd vaccine doses. However, the phenotype was dominated by non-switched IgM/IgD B cells and resembled newly primed immune responses, including early plasmablasts, and after dose 3, activated IgM memory B cells. Vaccine-induced B cells included both WT Spike/RBD-specific and cross-reactive Omicron BA1.1-specific B cells. After BTI with the Omicron VOC, the expansion of cross-reactive B cells correlated with increased serological IgG-anti-RBD responses, and vaccine-induced B cells were expanded and had isotype switched to IgG. In contrast, vaccine non-related Omicron-BA1.1 RBD-only specific and nucleocapsid-specific B cells had newly primed IgD^+^ phenotype. BTI in KTR had significantly increased systemic inflammatory markers that included innate immune activation (MPO, Calprotectin, LBP) and pro-inflammatory cytokines (IL-18, IL-6, IP-10).

The cohort also included serological non-responders selected for 4th vaccination (interventional trial). These had a 10-fold lower median frequency of Spike-specific dextramer CD8 T cells, 25% had a barely detectable Spike-specific population, but CTL had an early effector phenotype (classical after vaccination). Despite the low frequency of B cells in this group, 34% developed detectable RBD- or Spike-binding B cells after dose 4 and we could detect activated RBD- or Spike-binding- IgG^+^ memory B cells. This suggested a delayed vaccine response in this group. Nevertheless, about 15% were global low or non-responders after dose 4. Further vaccination (doses 5, 6) resulted in incremental improvement of a two-fold BAU/mL ratio (D5m1/D4m1) in serological responses, even in previously non-responder patients.

In Norway, most patients receive a low level of immunosuppression since we dose CNI according to the findings from the Elite-Symphony trials with low tacrolimus levels (4–7 ng/ml) already from the time of transplantation^34^^,^^35^ that is used in most centres around the world and our standard MMF protocol is 750 mg BID. Despite similar immunosuppression therapy, the individual impacts of MPA, CNI type (cyclosporine vs tacrolimus), and glucocorticoids were not evaluable in the present selection of KTR and need to be further delineated. We can anticipate that triple drug recipients (CNI, MPA, and prednisolone) will more likely have no or low serological response,^20^ but lowering immunosuppression increase the risk of acute rejections in some patients and the development of donor-specific HLA antibodies (DSA) in others. Rather, we have continued vaccinations in non-responders and a few patients have currently (March 2023) received 8 doses (AÅ et al., Ms in prep).

We and others have demonstrated that KTR has reduced serological responses to SARS-CoV-2 vaccines and lower IgG anti-RBD after standard vaccination.^7^, ^8^, ^9^, ^10^, ^11^, ^12^, ^13^, ^14^, ^15^, ^16^, ^17^, ^18^, ^19^, ^20^ This already weak response could be alarming in light of successive VOC waves that can evade antibody protection^36^, ^37^, ^38^ and confer increased transmissibility.^39^^,^^40^ However, Spike-specific T cell immunity is less affected by mutations, and evidence thus far indicates that T cells play a critical role in protection against SARS-CoV-2.^41^ We found normal T-cell responses in most KTR, even in patients with low or negative serological responses (dose 3). T cells are necessary for rapid and efficient resolution of COVID-19,^41^, ^42^, ^43^ for protection against severe infection in settings of low antibody levels, and rapid viral control in the absence of antibodies–controlling infection in most but not all healthy individuals.^41^^,^^44^, ^45^, ^46^ Moreover, in contrast to decaying antibody titers, SARS-CoV-1 T cell memory was long-lasting, and specific T cell responses were still detected after 17 years.^47^

Solid organ transplant recipients that had received two SARS-CoV-2 vaccine doses had an 82-fold higher risk of a COVID-19 BTI and a 485-fold higher risk of hospitalization and death compared to fully vaccinated adults until April 2021 in the USA.^48^ Here, we described systemic inflammation induced by BTI despite the induction of SARS-CoV-2 specific T cells and B cells in non-hospitalized KTR. Further investigations are suggested to understand the interplay between innate and adaptive immunity in KTR and to what extent the observed inflammation boosts vaccine immunity. In this regard, Spike-specific CD8 T cells in vaccine responder patients were activated, obtained markers of effector function, did not express exhaustion or senescence markers, and displayed a matured phenotype after re-vaccination. After BTI, CD8 T cells specific for Spike as well as non-Spike expressed markers related to cytotoxic function (KLRG1^Hi^GPR56^Hi^CRACC^Hi^CD244^Hi^), as well as activation markers (CD27^+^PD-1^Low^HLA-DR^+^), but not exhaustion/senescence, demonstrating appropriate effector functions after BTI, despite CNI therapy. Similarly, vaccine-generated cross-reactive B cells expanded in the settings of Omicron infection and systemic inflammation, and Spike-specific B cells finally isotype switched to IgG and obtained memory markers. Thus, it is possible that the observed BTI-induced cytokines (e.g. IL-18, IL-6, IP-10) helped surmount a threshold that supported T and B cell differentiation as well as plasma cell development.^49^, ^50^, ^51^

In normal individuals, mRNA-based vaccination induces a robust but transient SARS-CoV-2-specific antibody response, and persistent SARS-CoV-2-specific GC B cell^52^, ^53^, ^54^ and T_FH_ cell response,^55^^,^^56^ as we also detected in the current controls (current results, previous work^23^ and Ms in preparation). In KTR, it was obvious that the germinal centre responses had been less productive as we found mostly newly activated Spike or RBD-binding B cells or plasmablasts that had not isotype class switched—suggesting delayed or stalled responses. In healthy donors, vaccination supports the development of Spike-specific Th cells and class-switched memory B cells that cross-react with Alpha, Beta, Delta, and Omicron RBDs and are capable of rapidly producing RBD binding antibodies.^23^^,^^33^^,^^53^ We demonstrated that the vaccination in KTR enabled the development of RBD and Spike-binding B cells that were further expanded after BTI in Omicron-infected KTR. Importantly, this viral recall of vaccine immunity correlated with IgG anti-RBD levels after BTI demonstrating the role of infection as a boost of vaccine immune responses. Nevertheless, the hybrid immunity in KTR also included de novo B cell priming and expansions of Omicron-RBD-binding, Omicron-Spike binding, or non-Spike binding B cells (not isotype switched).

Even though most patients had vaccine responses in one or more of the immune dimensions analysed, it was obvious that some of the patients failed to seroconvert after vaccination and therefore had no other immunological protection. The vulnerability of such global non-responders to succumb to COVID-19 remains unclear. Nevertheless, we found that each successive vaccination improved immunity in many patients. Considering the low cost and low levels of new side effects in the revaccination,^20^ we suggest that serological non-responders receive further revaccination doses. Vaccination could allow stepwise expansion of delayed and low-frequency SARS-CoV-2 specific T or B cells as shown here, to protect against current circulating VOC (XBB, BA.5, and BQ.1).

## Contributors

HK, AÅ, KM, and LAM conceived and designed the study. HK and LAM drafted the paper. HK, AA, KPL, LT, AC, TdMK, and GG performed experiments. HK, AA, KH, AC, LT, TdMK, MH, GG, JTV, FLJ, KM, AÅ, and LAM verified, analysed and interpreted underlying data; JRO, AVR, MK, and GLG contributed to data analysis and interpretation. KPR, ÅA, KM, and JTV organized a collection of samples and information from the cohorts. KH, KM, AVR, MH, and AÅ handled patients and clinical data processing. All authors contributed to and approved the final manuscript.

## Data sharing statement

The datasets generated and analysed during the current study are available from the corresponding author upon reasonable request. The patient samples are not available on request due to restricting ethical and legal approvals.

## Declaration of interests

GG has received speaker bureaus from Pfizer, Sanofi, GSK, Vitusapotek, Bayer, and ThermoFisher; consulting fee from Norsk Pasientskadeerstatning and advisory board for Moderna, Seqirus, and Janssen. GLG has received speaker bureaus from Novartis, AbbVie, and Pfizer. KH has received speaker bureaus and travel support from Astra Zeneca. AÅ has received the support of free study drug/placebo in investigator-initiated RCT from AstraZeneca and has participated in an advisory board for AstraZeneca. LAM has received speaker bureaus from Incyte Biosciences and Janssen and a fee for expert testimony from the Norwegian Medicines Agency. HK, AA, KPL, LT, TdMK, MK, MH, AVR, JTV, FLJ, and KM declare no competing interests.
