# Supplementary material for: Hybrid and SARS-CoV-2-vaccine immunity in kidney transplant recipients
Source: eBioMedicine. 2023 Oct 14;97:104833. doi: 10.1016/j.ebiom.2023.104833 (PMC10585642; doi:10.1016/j.ebiom.2023.104833)
Supplement: Supplementary Tables and Figs [file mmc1.pdf]

Supplementary Data and Materials

Hassen Kared, et al - Hybrid and SARS-CoV-2-vaccine immunity in kidney transplant recipients

<u>Inventory:</u>

Supplementary Materials

Supplementary Figures 1-7

Supplementary Figure S1. Study Design and survival curves

Supplementary Figure S2. Quantification of B cells in HD and KTR

Supplementary Figure S3. Analysis of SARS-CoV-2-specific B cells in KTR and HD

Supplementary Figure S4. Cytotoxic cellular immunity during vaccination and BTI in KTR

Supplementary Figure S5. T Helper cellular immunity during vaccination and BTI

Supplementary Figure S6. Integrative analysis of vaccinal response after the fourth dose

Supplementary Figure S7. Inflammation in KTR before vaccination (baseline) and after BTI.

Supplementary Tables

Supplementary Table 1 – Demographic data for cohorts

Supplementary Table S2. Peptide: HLA multimers

Supplementary Materials

Patients

See Supplementary Table S1 and Figure 1 for details on the KTR cohort. The following KTR characteristics were investigated; age, sex, previous mRNA vaccine type (Comirnaty vs. Spikevax), SARS-CoV-2 spike antibody level (log BAU/mL), use of immunosuppressive drugs (calcineurin inhibitors (CNI), mycophenolate (MPA), everolimus (EVR)), use of immunosuppressive combinations (CNI+MPA+prednisolone, CNI+prednisolone, other), years since the last transplant, years in renal replacement therapy, preemptive transplantation, recipient CMV serology (positive/negative), BMI (kg/m^2), and eGFR (mL/min/1.73 m^2). Information on vaccination was obtained from the Norwegian Immunization Registry (SYSVAK) and on infection from the Norwegian Renal Registry.

Sample preparation and HLA typing

Each sample consisted of two frozen aliquots with an average cell number of 10 million cells and viability above 95% per donor. Samples were thawed at 37°C and immediately transferred into a complete RPMI medium (10% FCS, 1% penicillin /streptomycin, glutamine, 10 mM HEPES). After the first wash, thawed cells were incubated for 15 minutes at room temperature with DNAse (STEMCELL). Live cells were purified by removing dead cells using a column-based magnetic depletion approach according to the manufacturer’s recommendations (Miltenyi). Vaccinated healthy donor PBMCs matched for at least one of the donor HLA alleles were included in each experiment as a control for specific T cell identification. VeriCells were included in each experiment as a control for phenotypic markers.

The peptides listed below are referenced individually in the Supplementary Section. Spike Specific CTL were detected using PE-conjugated Dextramers (Immudex) targeting Spike and restricted to HLA-A*0101 (LTDEMIAQY), HLA-A*0201 (YLQPRTFLL), HLA-A*2402 (QYIKWPWYI), and HLA-B*0702 (SPRRARSVA). The panel was expanded using Flex-T tetramer according to the manufacturer´s instructions (BioLegend). We UV-exchanged peptides for Spike epitopes restricted to HLA-A*0101 (YTNSFTRGVY), HLA-A*0201 (LITGRLQSL and RLNEVAKNL), HLA-A*2402 (NYNYLYRLF), and HLA-B*0702 (APHGVVFL) and tetramerized with Streptavidin-PE (Biolegend). A similar approach was performed for non-Spike derived epitopes, including HLA-A*0101 (ORF3a, FTSDYYQLY, and ORF1ab, TTDPSFLGRY), HLA-A*0201 (ORF3a, LLYDANYFL), HLA-A*2402 (ORF3a, VYFLQSINF), and HLA-B*0702 (Nucleoprotein, SPRWYFYYL) and tetramerized with Streptavidin-APC (Biolegend). CMV-and EBV/FLU-specific CD8 T cells were generated similarly and tetramerized using Streptavidin-PECF594 (Biolegend) and Streptavidin-PE-Cy5 respectively. CMV-derived epitopes were for HLA-A*0101 (DNA polymerase processivity factor, VTEHDTLLY), HLA-A*0201 (65 kDa phosphoprotein, NLVPMVATV), HLA-A*2402 (65 kDa phosphoprotein, QYDPVAALF), and HLA-B*0702 (65 kDa phosphoprotein, RPHERNGFTVL) and EBV derived epitopes were for HLA-A*0201 (EBV LMP2, FLYALALLL) and HLA-B*0702 (EBV antigen 3, RPPIFIRRL). A Flu peptide was for HLA-A*0101 (Nucleoprotein, Influenza A virus CTELKLSDY). All peptides were ordered from Genscript with a purity above 85% by HPLC purification and mass spectrometry. Lyophilized peptides were reconstituted at a stock concentration of 10 mM in DMSO.

Antigen-specific multimer CD8 T cells were identified by fine manual gating, as described (*1*). The designation of bona fide antigen-specific T cells was further dependent on (a) the detection cut-off threshold (≥ 5 events to be detected), (b) the background noise (frequencies

of specific $CD8^+$ T cells must be greater than frequencies from the corresponding $CD4^+$ T cell population) as unbiased objective criteria for antigen-specificity assessment. Spike and non-Spike Dextramers staining have been extensively validated in COVID-19 convalescent patients and in SARS-CoV-2 vaccinated healthy donors during longitudinal follow-up.

Flow cytometry

MAbs and stains were for BD FACSymphony: BB515 Mouse Anti-Human CD279 (PD-1) Clone EH12.1, BD Biosciences, PerCP-eFluor 710, KLRG1 Monoclonal Antibody (13F12F2), eBioscience, PE/Cyanine7 anti-human GPR56, Clone CG4, Nordic Biosite, Alexa Fluor 700 anti-human CD244 (2B4), clone C1.7, Nordic Biosite, APC/Cyanine7 anti-human HLA-DR, clone L243, Nordic Biosite, BV480 Rat Anti-Human CXCR5 (CD185) (Clone: RF8B2) BD Biosciences, BB515 Mouse Anti-Human CD38, clone , HIT2 BD Biosciences, Brilliant Violet 570™ anti-human CD3, Nordic Biosite, Brilliant Violet 605, CD127 Mouse anti Human, Clone HIL 7R M21, BD Biosciences, Brilliant Violet 650, CD161 Mouse anti Human, clone: DX12, BD Biosciences, BV711 Mouse Anti-Human TIM-3 (CD366), clone 7D3, BD Biosciences, BV750 Mouse Anti-Human CD8, clone SK1, BD Biosciences, Brilliant Violet 785™ anti-human CD57 Recombinant, clone QA17A04, Nordic Biosite, BV421 Mouse Anti-Human CD319 (CRACC), BD Biosciences, BUV395 Mouse Anti-Human TIGIT, clone 741182, BD Biosciences, Live/dead™ Fixable Blue Dead Cell Stain Kit, for UV excitation, Thermo Fisher Scientific, BUV563 Mouse Anti-Human CD45RO, clone UCHL1, BD Biosciences, BUV615 Mouse Anti-Human CD95, clone DX, BD Biosciences, BUV661 Mouse Anti-Human CD4, clone SK3, BD Biosciences, BUV737 Mouse Anti-Human CD38, clone HB7, BD Biosciences, BUV805 Mouse Anti-Human CD27, clone L128, BD Biosciences. VeriCells PBMC (BioLegend) were included as controls.

Frequency values were calculated based on the percentage of the parent immune cell population and phenotypic markers were gated individually for each sample and calculated as % of positive cells. High-dimensional phenotypic profiles and sample distributions were shown using uniform manifold approximation and projection. Data analysis was performed using CYTOGRAPHER® (ImmunoScape cloud-based analytical software), custom R-scripts, GraphPad Prism (GraphPad Software), and FlowJo v10 software (BD Life Sciences). Statistical significance was set at a threshold of $*p < 0.05$, $**p < 0.01$, and $***p < 0.001$.

In vitro stimulation assays

Thawed cells were stimulated for 16h with SARS-CoV-2 PepTivator Spike protein peptides consisting of 15-mer sequences with 11 amino acid overlaps (Wuhan-Hu-1, i.e. wild type WT. Miltenyi Biotec) as described (*1*), Peptide stimulation was performed on 1 million PBMCs per condition in the presence of costimulatory antibodies against CD28 and CD49d (BD Biosciences) and Brefeldin-A (10 µg/mL, Millipore Sigma). SARS-CoV-2-specific T cells were identified by dual expression of CD40L (CD154) and CD137, interferon-gamma (IFN-γ), interleukin-2 (IL-2), or tumor necrosis factor (TNF) for $CD4^+$ T cells and by dual expression of IFN-γ and TNF or CD137 and IL-2, TNF or IFN-γ for $CD8^+$ T cells.

Inflammatory markers

The following enzyme-linked immunosorbent assay (ELISA) kits were used according to manufacturer protocols. From R&D Systems: Human CD14 DuoSet ELISA (DY383), Human CD163 DuoSet ELISA (DY1607), Human LBP DuoSet ELISA (DY870-05), Human Galectin-9 DuoSet ELISA (DY2045), Human GDF-15 Quantikine ELISA Kit (DGD150), Human CXCL4/PF4 Quantikine ELISA (Kit DPF40), Human IFN-alpha (41100); from

Ebioscience: Human MPO Instant ELISA Kit (BMS2038INST); from Thermo Scientific: Invitrogen novex IP 10 Human ELISA Kit (KAC2361); from Abcam Human C-Reactive Protein/CRP (Ab99995); from MyBioSource: Human zonulin ELISA Kit (MBS706368); from Meso Scale diagnostics: human Calprotectin (F21YB-3) (*2*).

Measurement of neutralizing antibodies

Vero E6 cells were added into 96-well plates (Costar 3595, Corning Incorporated) in $1x10^4$ cells/well. The next day, titrated amounts of sera that had been inactivated at 56°C for 30 min were mixed with TCID100 of SARS-CoV-2 viruses (either Human 2019-nCOV strain 2019-mCoV/Italy-INM1 – provided by the European Virus Archive GLOBAL (EVA-GLOBAL that has received funding from the European UnioWs Horizon 2020 program under grant agreement No $71029), SARS-CoV-2/Norway/11421/2021 (Delta/B.1.617.2), or SARS-CoV-2/Norway/29450/2021 (Omicron/B.1.1.529) in triplicates. Following 1.5 hours of incubation, the mixtures were added to the cells and incubated for 4 days at 37ºC in a 5% CO_2 humidified atmosphere. Next, the plates were washed with PBS and fixed with acetone/PBS for 30 min. The plates were air-dried, washed, and incubated with rabbit anti-SARS-CoV-2 nucleocapsid antibody (cat. 40143-R004, Sino Biological) overnight at 4°C. Plates were incubated with horseradish peroxidase (HRP)-conjugated anti-rabbit IgG Fc antibody (cat. SSA003, Sino Biological) for 1 hour at room temperature, developed with TMB Substrate Solution (cat. N301, ThermoFisher), stopped with 1 M of hydrochloric acid, and read with an EnVision 2104 Multilabel Reader (Perkin Elmer). The neutralization titer was determined as the highest plasma dilution that neutralized more than 50% of the virus. The assay has been validated by comparison with other laboratories (*3*).

Supplementary Figures

Supplementary Figure S1. Study Design and survival curves

a. Study design to evaluate COVID-19 vaccine efficiency in kidney transplanted patients. The KTR with no detectable serological response after the third dose of vaccine were included in the interventional trial arm for further immune analysis. The serological response was monitored in the observational cohort for later vaccine administrations. Both cohorts were followed up to identify SARS-CoV-2 VOCs infection in vaccinated KTR.

b. Pooled IgG anti-RBD level (BAU/mL) for interventional trial and observational trial from KTR vaccinated in the standard Norwegian Corona Vaccination Program. Doses are indicated as in a., and further before dose 5 (D5), 1 mo after dose 5 (D5m1), after dose 6 (D6), and 1 mo after dose 6 (D6m1).

c. Normal population vaccinated in the standard Norwegian Corona Vaccination Program. IgG anti-RBD level is shown for 1-6 mo after dose 2 (D2m1-6), 6-9 mo after dose 2 (D2m6-9), before dose 3 (D3), 1-6 mo after dose 3 (D3m1-6), 6-9 mo after dose 3 D3m6-9. Two-tailed Wilcoxon matched-pairs signed rank test.

d. Anti-SARS-CoV-2 RBD IgG decay after fourth dose of vaccine in KTR. Paired KTR samples were analyzed one month after D4 and just before receiving dose 5 of the COVID-19 vaccine (n=60). Two-tailed Wilcoxon matched-pairs signed rank test.

e. Timeline of cumulative SARS-CoV-2 infection in KTR. The prevalence of infection was represented by year, independently of the circulating variant of concern, and by the administered vaccine dose.

f. Kaplan Meier overall graft- and g. death- censored graft survival analysis of the entire Norwegian Kidney transplanted patient cohort alive with a functioning graft by February 24th,

2020 (n=3620), that during the pandemic (through October 2022) was reported with SARS-CoV-2 infection to the Norwegian Renal Registry. The analysis is grouped by COVID severity, i.e., infection without- or with hospitalization, and transferred to the ICU department. The log-rank statistical test result is shown in the figure.

Supplementary Figure S2. Quantification of B cells in HD and KTR

a. Frequency of B cells that bind Spike but not RBD ($Spike^{+}RBD^{-}$ B cells) or bind Spike- and RBD ($Spike^{+}RBD^{+}$ B cells) in total B cells before vaccination (baseline, BL), 1 mo after dose 1 (D1m1), 1 mo after dose 2 (D2m1), 6 mo after dose 2 (D2m6), 1 mo after dose 3 (D3m1) (n=16).

b. Phenotype of HD responses for time points as in a. visualized by principal component analysis (PCA) biplots of selected markers as indicated (arrows) (n=16).

c. Percentage of Spike-binding B cells that are positive for the markers as indicated (IgM^{+}, IgG^{+}, $CD21^{+}$, $IRF\text{-}1^{+}$, $CD71^{+}$, or $CD138^{+}$) 1 mo after dose 2 or dose 3 (n=16).

d. Visualization by an UMAP-plot for Spike-binding B cells in KTR for the markers shown in c. Phenograph clusters (left) and corresponding UMAP at D2m1 (n=24) and D3m1 (n=23) (right) are shown. Each dot represents one KTR donor.

e. Cluster distribution from e, the frequency of B cells in each cluster is shown in KTR at D2m1 and D3m1.

f. Characterization of Spike-binding B cells after vaccination in KTR - markers in clusters from d and e. The normalized frequency of each marker is visualized by a cold-to-hot heat map and an automatic hierarchical clustering of Spike-binding B cells for each marker is shown.

Supplementary Figure S3. Analysis of SARS-CoV-2 specific B cells in KTR and HD

a. Left panels: Eight representative examples of B cells from HD that bind BA1.1 (Omicron VOC) Spike and WT Spike. Cross-reactive B cells that bind both (top region), public Spike in text, or WT Spike only (bottom region) are shown. Right panels: Eight representative examples of B cells from HD that bind BA1.1 (Omicron VOC) RBD and WT RBD. Cross-reactive B cells that bind both (top right region), public RBD in text or WT RBD only (bottom right region), or BA1.1 RBD only (top left region) are shown.

b. Left panels: Four representative examples of B cells from KTR before and after BTI, BA1.1 Spike vs WT Spike dot plots are shown. Right panels: Four representative examples of B cells from KTR that bind BA1.1 (Omicron VOC) RBD and WT RBD. Regions: Public RBD, WT RBD only (bottom right region), and BA1.1 RBD only (top left region) are shown.

c. Left panels: Four representative examples of B cells from KTR before and after BTI, dot plots show CD38 (activation) vs WT RBD. Right panels: Four representative examples of B cells from KTR before and after BTI, dot plots show CD71 (activation) vs WT RBD

d. Phenotypes of B cells from KTR after BTI. The expression of markers is described for B cells that bind RBD or Spike (BA1.1 or Public or WT) with automated hierarchical clustering of the markers (n=16).

e. Quantification of individualized markers for B cells after BTI. Violin plots described B cells that bind specific or cross-reactive epitopes (BA1.1 or Public or WT), top as indicated, or that bind Nucleocapsid, RBD, or Spike (n=25).

f. Viral neutralization assay for WT, Delta, and Omicron VOC for HD after 3 doses (n=7), KTR before vaccination (n=13), and KTR BTI (n=7),. Two-tailed Wilcoxon paired test, $p<0.0001$.

Figure S4. Cytotoxic cellular immunity during vaccination and BTI in KTR.

a. Quantification of SARS-CoV-2 Spike-specific CD8 T cells defined by HLA restriction in vaccinated HD (n=37) and KTR (n=25) after dose 2.

b. Quantification of SARS-CoV-2 Spike-specific CD8 T cells in vaccinated KTR after dose 2 (n=25) and dose 3 (n=12).

c. Visualization by an UMAP-plot of T cell markers (KLRG1, CD160, CD244, HLA-DR, CRACC, CD127, TIGIT, CD95, CD27, CD45RO, GPR56, and PD-1) in SARS-CoV-2 Spike and CMV-specific CD8 T cells. The clusters were automatically identified by phenograph software. SARS-CoV-2 Spike and CMV-specific CD8 T cells are visualized in green and red respectively.

d. Characterization of SARS-CoV-2 Spike-specific CD8 T cells in vaccinated HD. A cold-to-hot heatmap represents the scaled frequency of each marker expressed by antigen-specific $CD8^+$ T cells. The frequency of each marker is displayed, and the automatic hierarchical clustering of markers expressed by HLA-class I restricted multimers and of patients is shown. The top four rows indicate the HLA for A and B alleles, epitope-derived viruses, age, and frequencies, (n=37).

e. Phenotype of virus-specific CD8 T cells in vaccinated HD visualized by principal component analysis (PCA) biplots of selected markers as indicated (arrows).

f. Visualization by an UMAP-plot of selected markers (described in c.) in virus-specific CD8 T cells from BTI in KTR.

g. Distribution of markers from virus-specific CD8 T cells in KTR after BTI. Correlogram describes co-expression or exclusion between molecules at the surface of Spike, non-Spike, CMV, EBV, and FLU-specific CD8 T cells.

Figure S5. T Helper cellular immunity during vaccination and BTI.

a. Longitudinal follow-up of functional responses of SARS-CoV-2 specific T cells in healthy donors one month after the different doses of vaccine (n=16). Spike-specific CD4 T cells were identified by the up-regulation of activation markers such as CD154 and effector cytokines (TNF, IFN-γ, or IL-2). Spike-specific CD8 T cells were identified by the up-regulation of activation markers such as CD137 and TNF, IFN-γ, or Granzyme B by CD8 T cells.

b. Longitudinal follow-up of functional responses of SARS-CoV-2 specific T cells in KTR one month after the different doses of the vaccine (n=10). Spike-specific T cells were identified as in a.

c. Characterization of functional responses of SARS-CoV-2 specific CD4 T cells in KTR one month after BTI (n=15). Wuhan or mutated Spike-, and non-Spike-specific CD4 T cells were identified as in a.

d. Longitudinal follow-up of functional responses of SARS-CoV-2 specific CD4 T cells in KTR before and after BTI (n=8). Samples are analyzed for Spike- and MNO (non-Spike)-specific CD4 T cells ($CD154^{+}CD137^{+}$).

Supplementary Figure S6 Integrative analysis of vaccinal response after the fourth dose

Quantification of vaccine responsiveness after D4. Pie charts represented the frequency of KTR with RBD IgG seroconversion (threshold at 2000 BAU/mL) and with anti-Spike SARS-CoV-2 cellular response (threshold at 0.01% of specific CD4 or CD8 T cell response), n=58.

Supplementary Figure S7 Inflammation before vaccination and after BTI in KTR

a. Systemic inflammatory profile of KTR before vaccination. ELISAs were performed on the plasma of HD (n=20) and KTR (n=13). Statistical differences are indicated in the graph.

b. Inflammatory signature of KTR before vaccination. The specific distribution of pro-inflammatory cytokines is displayed in PCA biplots (see Methods). KTR (orange) and HD (blue) were identified by automatically generated ellipses.

c. Systemic inflammatory profile of KTR after BTI. ELISAs were performed on the plasma of BTI HD (n=13) and BTI KTR (n=12). Statistical differences are indicated in the graph.

d. Inflammatory signature of KTR after BTI. The specific distribution of pro-inflammatory cytokines is displayed in PCA biplots (see Methods). KTR (red) and HD (dark blue) were identified by automatically generated ellipses.

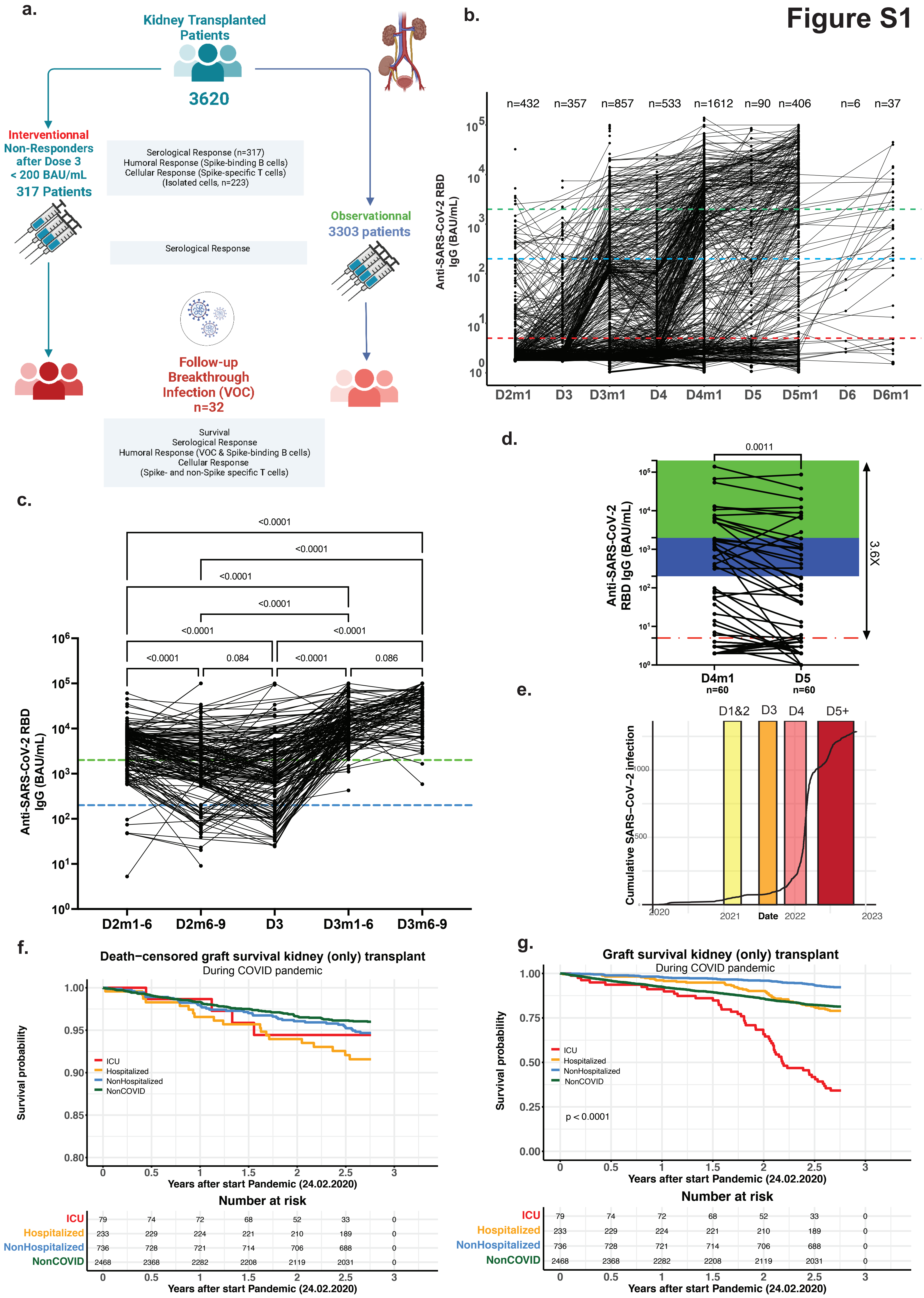
a.
Kidney Transplanted Patients
3620
Interventionnal Non-Responders after Dose 3 < 200 BAU/mL
317 Patients
Serological Response (n=317)
Humoral Response (Spike-binding B cells)
Cellular Response (Spike-specific T cells)
(Isolated cells, n=223)
Serological Response
Observationnal
3303 patients
Follow-up Breakthrough Infection (VOC)
n=32
Survival
Serological Response
Humoral Response (VOC & Spike-binding B cells)
Cellular Response
(Spike- and non-Spike specific T cells)
b.
n=432 n=357 n=857 n=533 n=1612 n=90 n=406 n=6 n=37
Anti-SARS-CoV-2 RBD IgG (BAU/mL)
D2m1 D3 D3m1 D4 D4m1 D5 D5m1 D6 D6m1
c.
<0.0001
0.084
0.086
Anti-SARS-CoV-2 RBD IgG (BAU/mL)
D2m1-6 D2m6-9 D3 D3m1-6 D3m6-9
d.
0.0011
Anti-SARS-CoV-2 RBD IgG (BAU/mL)
3.6X
D4m1 n=60
D5 n=60
e.
D1&2 D3 D4 D5+
Cumulative SARS-CoV-2 infection
1000
500
2020 2021 Date 2022 2023
f.
Death-censored graft survival kidney (only) transplant
During COVID pandemic
Survival probability
ICU
Hospitalized
NonHospitalized
NonCOVID
Years after start Pandemic (24.02.2020)
Number at risk
ICU 79 74 72 68 52 33 0
Hospitalized 233 229 224 221 210 189 0
NonHospitalized 736 728 721 714 706 688 0
NonCOVID 2468 2368 2282 2208 2119 2031 0
g.
Graft survival kidney (only) transplant
During COVID pandemic
Survival probability
p < 0.0001
ICU
Hospitalized
NonHospitalized
NonCOVID
Years after start Pandemic (24.02.2020)
Number at risk
ICU 79 74 72 68 52 33 0
Hospitalized 233 229 224 221 210 189 0
NonHospitalized 736 728 721 714 706 688 0
NonCOVID 2468 2368 2282 2208 2119 2031 0

Figure S1

Figure S2

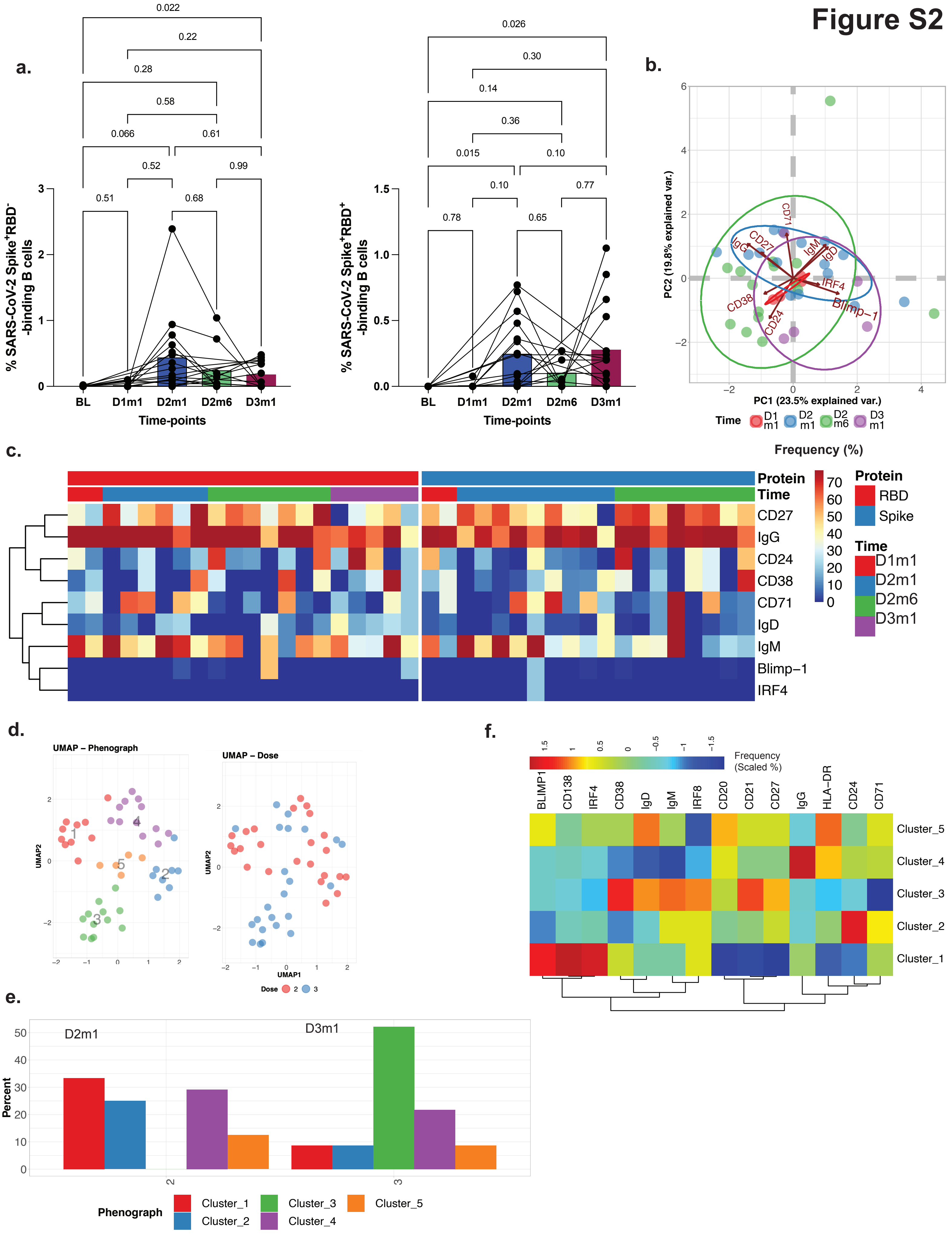
a.
% SARS-CoV-2 Spike+RBD- -binding B cells
% SARS-CoV-2 Spike+RBD+ -binding B cells
Time-points
BL
D1m1
D2m1
D2m6
D3m1
b.
PC1 (23.5% explained var.)
PC2 (19.8% explained var.)
Time
c.
Frequency (%)
Protein
Time
RBD
Spike
CD27
IgG
CD24
CD38
CD71
IgD
IgM
Blimp−1
IRF4
d.
UMAP – Phenograph
UMAP – Dose
UMAP1
UMAP2
Dose
e.
Percent
Phenograph
Cluster_1
Cluster_2
Cluster_3
Cluster_4
Cluster_5
f.
Frequency (Scaled %)
BLIMP1
CD138
IRF4
CD38
IgD
IgM
IRF8
CD20
CD21
CD27
IgG
HLA-DR
CD24
CD71

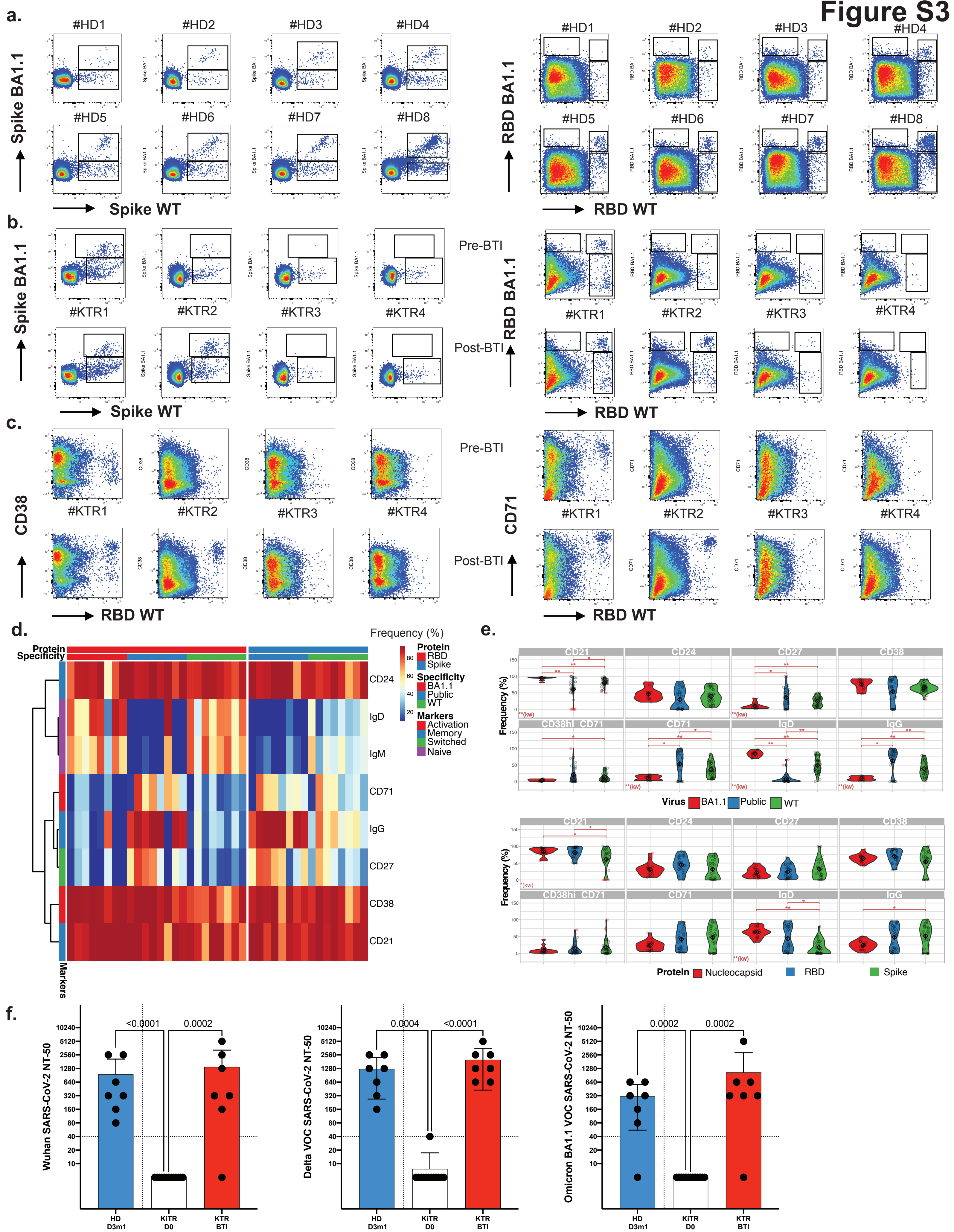
Figure S3
a.
#HD1
#HD2
#HD3
#HD4
#HD5
#HD6
#HD7
#HD8
Spike BA1.1
Spike WT
RBD BA1.1
RBD WT
b.
#KTR1
#KTR2
#KTR3
#KTR4
Pre-BTI
Post-BTI
c.
CD38
CD71
d.
Protein
Specificity
Frequency (%)
Protein
RBD
Spike
Specificity
BA1.1
Public
WT
Markers
Activation
Memory
Switched
Naive
CD24
IgD
IgM
CD71
IgG
CD27
CD38
CD21
Markers
e.
Frequency (%)
CD21
CD24
CD27
CD38
CD38hi_CD71
CD71
IgD
IgG
Virus
BA1.1
Public
WT
Protein
Nucleocapsid
RBD
Spike
f.
Wuhan SARS-CoV-2 NT-50
Delta VOC SARS-CoV-2 NT-50
Omicron BA1.1 VOC SARS-CoV-2 NT-50
<0.0001
0.0002
0.0004
<0.0001
0.0002
0.0002
HD D3m1
KiTR D0
KTR BTI

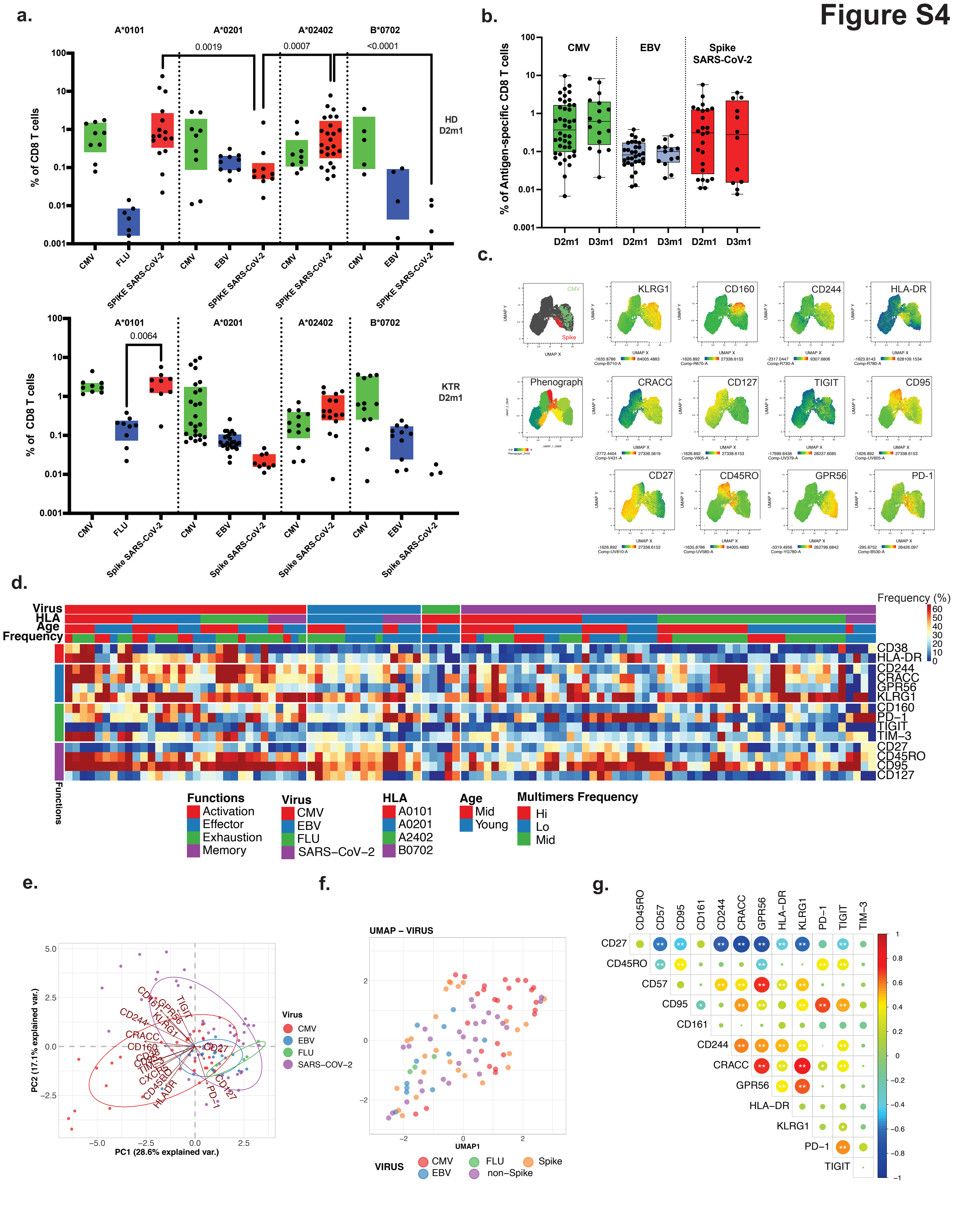
Figure S4
a.
A*0101
A*0201
A*02402
B*0702
0.0019
0.0007
<0.0001
% of CD8 T cells
HD
D2m1
CMV
FLU
SPIKE SARS-CoV-2
EBV
0.0064
KTR
D2m1
Spike SARS-CoV-2
b.
CMV
EBV
Spike
SARS-CoV-2
% of Antigen-specific CD8 T cells
D2m1
D3m1
c.
KLRG1
CD160
CD244
HLA-DR
Phenograph
CRACC
CD127
TIGIT
CD95
CD27
CD45RO
GPR56
PD-1
d.
Virus
HLA
Age
Frequency
Frequency (%)
CD38
HLA-DR
CD244
CRACC
GPR56
KLRG1
CD160
PD−1
TIGIT
TIM−3
CD27
CD45RO
CD95
CD127
Functions
Activation
Effector
Exhaustion
Memory
Virus
CMV
EBV
FLU
SARS−CoV−2
HLA
A0101
A0201
A2402
B0702
Age
Mid
Young
Multimers Frequency
Hi
Lo
Mid
e.
PC1 (28.6% explained var.)
PC2 (17.1% explained var.)
Virus
CMV
EBV
FLU
SARS−COV−2
f.
UMAP – VIRUS
UMAP1
VIRUS
CMV
EBV
FLU
non−Spike
Spike
g.
CD27
CD45RO
CD57
CD95
CD161
CD244
CRACC
GPR56
HLA−DR
KLRG1
PD−1
TIGIT
TIM−3

Figure S5

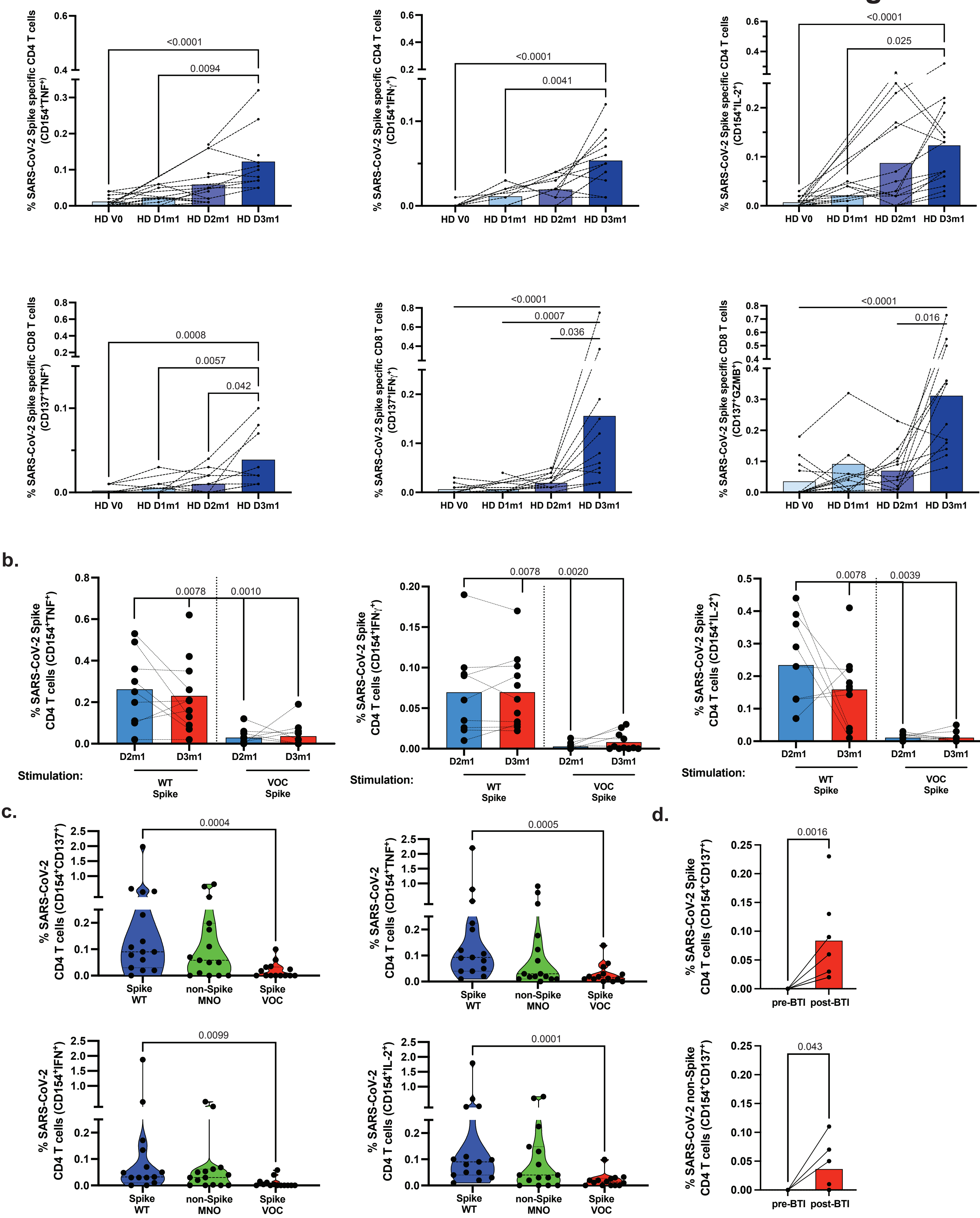

a.
% SARS-CoV-2 Spike specific CD4 T cells (CD154+TNF+)
% SARS-CoV-2 Spike specific CD4 T cells (CD154+IFNγ+)
% SARS-CoV-2 Spike specific CD4 T cells (CD154+IL-2+)
% SARS-CoV-2 Spike specific CD8 T cells (CD137+TNF+)
% SARS-CoV-2 Spike specific CD8 T cells (CD137+IFNγ+)
% SARS-CoV-2 Spike specific CD8 T cells (CD137+GZMB+)
HD V0
HD D1m1
HD D2m1
HD D3m1
<0.0001
0.0094
0.0041
0.025
0.0008
0.0057
0.042
0.0007
0.036
0.016
b.
% SARS-CoV-2 Spike CD4 T cells (CD154+TNF+)
% SARS-CoV-2 Spike CD4 T cells (CD154+IFNγ+)
% SARS-CoV-2 Spike CD4 T cells (CD154+IL-2+)
D2m1
D3m1
Stimulation:
WT Spike
VOC Spike
0.0078
0.0010
0.0020
0.0039
c.
% SARS-CoV-2 CD4 T cells (CD154+CD137+)
% SARS-CoV-2 CD4 T cells (CD154+TNF+)
% SARS-CoV-2 CD4 T cells (CD154+IFN+)
% SARS-CoV-2 CD4 T cells (CD154+IL-2+)
Spike WT
non-Spike MNO
Spike VOC
0.0004
0.0005
0.0099
0.0001
d.
% SARS-CoV-2 Spike CD4 T cells (CD154+CD137+)
% SARS-CoV-2 non-Spike CD4 T cells (CD154+CD137+)
pre-BTI
post-BTI
0.0016
0.043

Figure S6

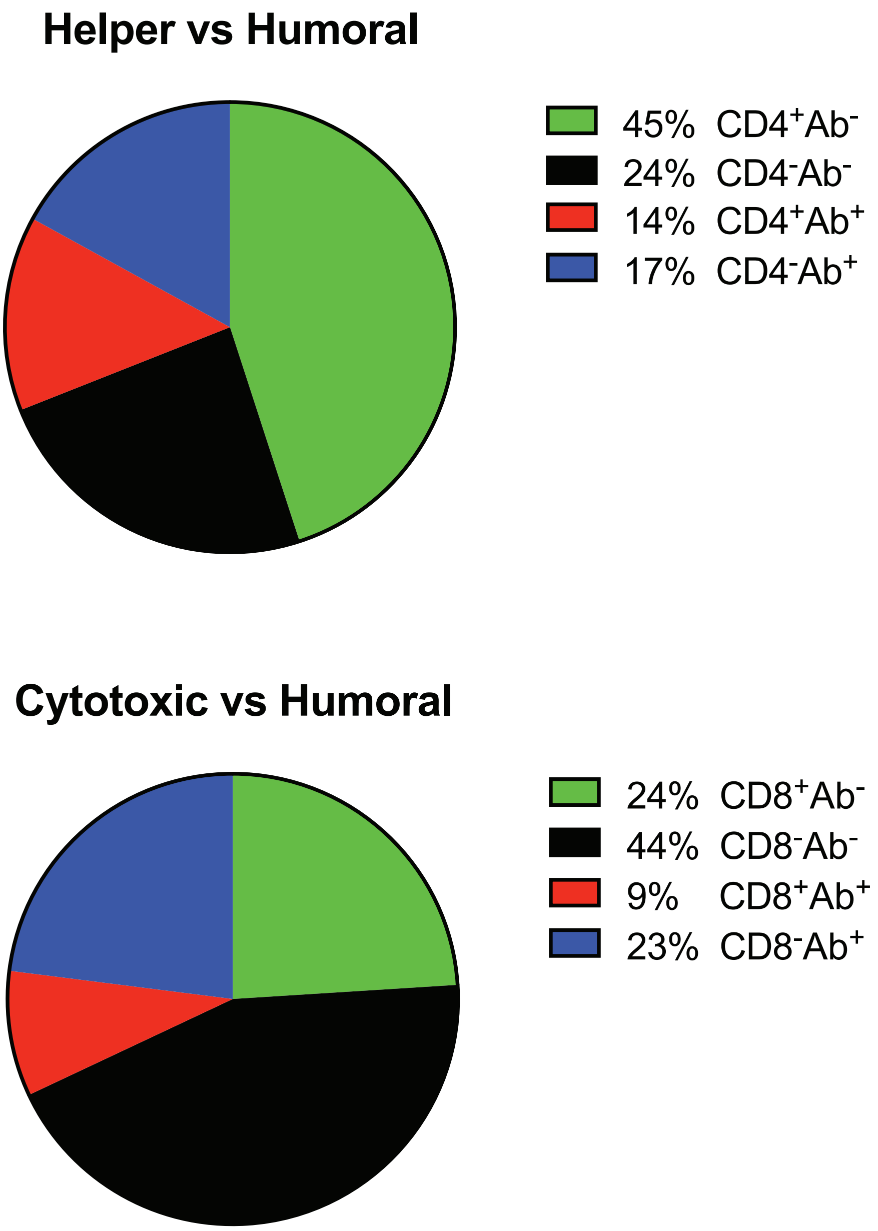
Helper vs Humoral
45% CD4+Ab-
24% CD4-Ab-
14% CD4+Ab+
17% CD4-Ab+
Cytotoxic vs Humoral
24% CD8+Ab-
44% CD8-Ab-
9% CD8+Ab+
23% CD8-Ab+

Figure S7

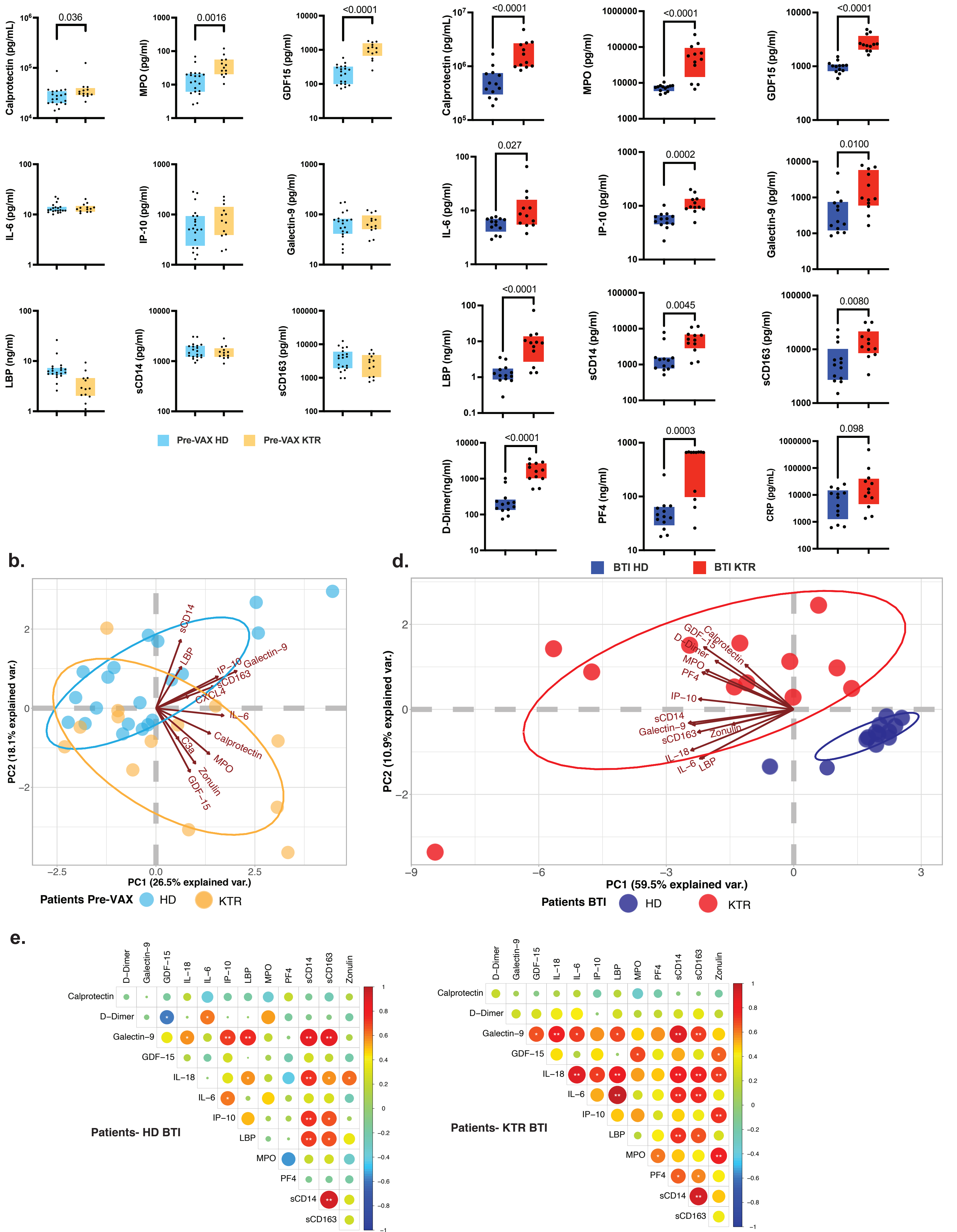

a.
Calprotectin (pg/mL)
0.036
MPO (pg/ml)
0.0016
GDF15 (pg/ml)
<0.0001
IL-6 (pg/ml)
IP-10 (pg/ml)
Galectin-9 (pg/ml)
LBP (ng/ml)
sCD14 (pg/ml)
sCD163 (pg/ml)
Pre-VAX HD
Pre-VAX KTR
c.
Calprotectin (pg/mL)
<0.0001
MPO (pg/ml)
<0.0001
GDF15 (pg/ml)
<0.0001
IL-6 (pg/ml)
0.027
IP-10 (pg/ml)
0.0002
Galectin-9 (pg/ml)
0.0100
LBP (ng/ml)
<0.0001
sCD14 (pg/ml)
0.0045
sCD163 (pg/ml)
0.0080
D-Dimer(ng/ml)
<0.0001
PF4 (ng/ml)
0.0003
CRP (pg/mL)
0.098
BTI HD
BTI KTR
b.
PC1 (26.5% explained var.)
PC2 (18.1% explained var.)
sCD14
LBP
IP-10
Galectin-9
sCD163
CXCL4
IL-6
Calprotectin
C3a
MPO
Zonulin
GDF-15
Patients Pre-VAX
HD
KTR
d.
PC1 (59.5% explained var.)
PC2 (10.9% explained var.)
GDF-15
Calprotectin
D-Dimer
MPO
PF4
IP-10
sCD14
Galectin-9
sCD163
Zonulin
IL-18
IL-6
LBP
Patients BTI
HD
KTR
e.
Calprotectin
D-Dimer
Galectin-9
GDF-15
IL-18
IL-6
IP-10
LBP
MPO
PF4
sCD14
sCD163
Zonulin
Patients- HD BTI
Patients- KTR BTI

Supplementary Table S1 – **Demographic data for cohorts. Mean (SD) or numbers (%)**

	Interventional trial (all)	Interventional trial - Isolated cells	All alive with functioning graft per 24.02.2020	COVID patients (up to 30.11.2022)	Non-COVID patients (up to 30.11.2022)
N	317	223	3620	1102	2518
Age	57.7 (13.0)	51.5 (18.4)	58.0 (14.7)	54.7 (14.3)	59.5 (14.7)
Male sex	188 (59%)	138 (62%)	2323 (64%)	675 (61%)	1648 (65%)
Years since last Tx	8.7 (7.2)	8.1 (7.0)	10.3 (8.3)	9.6 (8.0)	10.6 (8.5)
CNI+MPA+pred	264 (83%)	187 (84%)	2757 (76%)	877 (80%)	1880 (75%)
CNI+pred	17 (5%)	11 (5%)	351 (10%)	83 (8%)	268 (11%)
Other comb/missing	36 (11%)	25 (11%)	512 (14%)	142 (13%)	370 (15%)
CNI	288 (91%)	202 (91%)	3374 (93%)	1040 (94%)	2334 (93%)
MPA	292 (92%)	207 (93%)	2977 (82%)	944 (86%)	2033 (81%)
Prednisolone	314 (99%)	222 (100%)	3545 (98%)	1083 (98%)	2462 (98%)
mTORi	26 (8%)	21 (9%)	236 (7%)	61 (6%)	175 (7%)
Azathioprine	2 (1%)	1 (0.5%)	175 (5%)	51 (5%)	124 (5%)
Belatacept	1 (0.3%)	0 (0%)	13 (0.4%)	7 (1%)	6 (0.2%)
Missing	0 (0%)	0 (0%)	13 (0.4%)	2 (0.2%)	11 (0.4%)
eGFR (mL/min/1.73 m^2)	53 (19)	52 (18)	53 (22)	52 (21)	53 (23)

Supplementary Table S2. Peptide: HLA multimers

	HLA-A0201	HLA-A2402	HLA-B702	HLA-A0101	HLA-A1101
Spike	YLQPRTFLL<br>RLNEVAKNL<br>LITGRLQSL	QYIKWPWYI<br>NYNYLYRLF	SPRRARSVA<br>APHGVVFL	LTDEMIAQY<br>YTNSFTRGVY	
Non-'Spike	LLYDANYFL ORF3a 139-147	VYFLQSINF ORF3a 114-122	SPRWYFYYL Nucleocapsid 105-113	FTSDYYQLY ORF3a 207-215 and TTDPSFLGRY ORF1ab 1637-1646	KTFPPTEPK Nucleocapsid 362-370
CMV<br>EBV<br>FLU	NLVPMVATV<br>FLYALALLL	QYDPVAALF	RPHERNGFTVL<br>RPPIFIRRL	VTEHDTLLY<br><br>CTELKLSDY	

References

1. H. Kared *et al.*, Immune responses in Omicron SARS-CoV-2 breakthrough infection in vaccinated adults. *Nat Commun* **13**, 4165 (2022).
2. S. Holm *et al.*, Immune complexes, innate immunity, and NETosis in ChAdOx1 vaccine-induced thrombocytopenia. *Eur Heart J* **42**, 4064-4072 (2021).
3. D. Nguyen *et al.*, SARS-CoV-2 neutralising antibody testing in Europe: towards harmonisation of neutralising antibody titres for better use of convalescent plasma and comparability of trial data. *Euro Surveill* **26**, (2021).
